# Truncatenolide, a Bioactive Disubstituted Nonenolide Produced by *Colletotrichum truncatum*, the Causal Agent of Anthracnose of Soybean in Argentina: Fungal Antagonism and SAR Studies

**DOI:** 10.1021/acs.jafc.2c02502

**Published:** 2022-08-04

**Authors:** Marco Masi, Stefany Castaldi, Francisco Sautua, Gennaro Pescitelli, Marcelo Anibal Carmona, Antonio Evidente

**Affiliations:** † Dipartimento di Scienze Chimiche, Università di Napoli Federico II, Complesso Universitario Monte S. Angelo, Via Cintia 4, 80126 Napoli, Italy; ‡ Dipartimento di Biologia, Università di Napoli Federico II, Complesso Universitario Monte S. Angelo, Via Cintia 4, 80126 Napoli, Italy; § Cátedra de Fitopatología, Facultad de Agronomía, Universidad de Buenos Aires, C1417DSE Buenos Aires, Argentina; ∥ Dipartimento di Chimica e Chimica Industriale, Università di Pisa, Via Moruzzi 13, 56124 Pisa, Italy

**Keywords:** soybean, fungal diseases, Colletotrichum truncatum, bioactive nonenolides, truncatenolide, fungal antagonisms, SAR

## Abstract

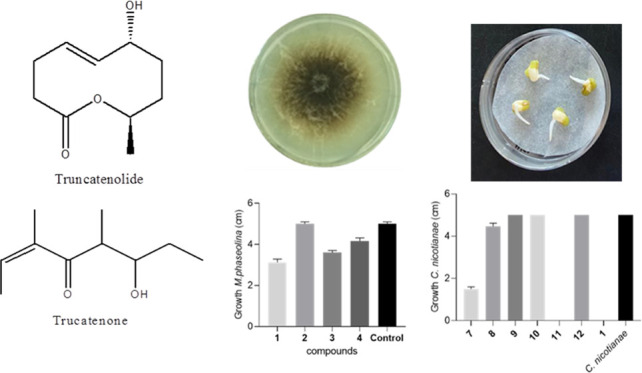

A bioactive disubstituted nonenolide, named truncatenolide, was produced by *Colletotrichum truncatum*, which was collected from infected tissues of soybean showing anthracnose symptoms in Argentina. This is a devastating disease that drastically reduces the yield of soybean production in the world. The fungus also produced a new trisubstituted oct-2-en-4-one, named truncatenone, and the well-known tyrosol and *N-*acetyltyramine. Truncatenolide and truncatenone were characterized by spectroscopic (essentially one-dimensional (1D) and two-dimensional (2D) ^1^H and ^13^C NMR and HR ESIMS) and chemical methods as (5*E,*7*R,*10*R*)-7-hydroxy-10-methyl-3,4,7,8,9,10-hexahydro-2*H*-oxecin-2-one and (*Z*)-6-hydroxy-3,5-dimethyloct-2-en-4-one, respectively. The geometry of the double bond of truncatenolide was assigned by the value of olefinic proton coupling constant and that of truncatenone by the correlation observed in the corresponding NOESY spectrum. The relative configuration of each stereogenic center was assigned with the help of ^13^C chemical shift and ^1^H–^1^H scalar coupling DFT calculations, while the absolute configuration assignment of truncatenolide was performed by electronic circular dichroism (ECD). When tested on soybean seeds, truncatenolide showed the strongest phytotoxic activity. Tyrosol and *N*-acetyltyramine also showed phytotoxicity to a lesser extent, while truncatenone weakly stimulated the growth of the seed root in comparison to the control. When assayed against *Macrophomina phaseolina* and *Cercospora nicotianae*, other severe pathogens of soybean, truncatenolide showed significant activity against *M. phaseolina* and total inhibition of *C. nicotianae*. Thus, some other fungal nonenolides and their derivatives were assayed for their antifungal activity against both fungi in comparison with truncatenolide. Pinolidoxin showed to a less extent antifungal activity against both fungi, while modiolide A selectively and totally inhibited only the growth of *C. nicotianae*. The SAR results and the potential of truncatenolide, modiolide A, and pinolidoxin as biofungicides were also discussed.

## Introduction

Soybean (*Glycine max*) (Linnaeus) Merrill, being a source of protein in foods and animal feeds, is considered one of the most important cultivated plants worldwide. Today, soybean is one of the most important crops in the world, the total market value of which was evaluated to be about US$146.23 billion in 2017.^1^ In fact, soybean is worldwide used as an essential raw product for foods, fuels, feeds, and biobased materials.^2−4^ This crop is produced mainly in the United States, Brazil, and Argentina.^5,6^

Considering the soybean biotic stress, the most severe are microbial diseases. Significant economic losses are induced by these diseases reported for many important arable vegetables, including soybean and fruit crops.^7,8^

These diseases are caused principally by bacteria and fungi, but the latter cause more significant losses in agrarian production. Typically, the foliar disease damage is less important, except for diseases like soybean rust, Pod blight, Rhizoctonia aerial blight or web blight, etc., which can cause severe losses when the weather conditions stimulate disease development.^9^

Charcoal rot on soybean is one of the deadliest diseases affecting this crop, caused by *Macrophomina phaseolina* (Tassi) Goidanich,^9^ a fungal pathogen hosted by about 500 cultivated and wild plants.^10^ To better understand the negative impact of *M. phaseolina* on soybean yield production, complex biology and genetic studies were carried out.^11−15^ Recently, the phytotoxins produced by *M. phaseolina* strain 2013-1 isolated in Argentina, which are potentially involved in charcoal rot disease, were investigated. The isolation and chemical characterization of two new phytotoxic penta- and tetrasubstituted cyclopentenones, named phaseocyclopentenones A and B and guignardone A, were reported.^16,17^

Soybean is extensively cultivated in Argentina, covering in the last few years 18 million hectares.^18^ This intensive cultivation has caused the introduction of different and severe diseases discussed above, which heavily affect the quantity and quality of the legume produced. This problem prompted the development of biocontrol strategies using seeds treated with biological control agents.^19^ Different bacteria were tested for their antifungal activity. Recently, two strains *Pseudomonas fluorescens* Migula 1895 (AL) 9 and *Bacillus subtilis* (Ehrenberg) Cohn 54 were selected^20^ and in greenhouse experiments they showed the most significant reduction in disease in soybean caused by *M. phaseolina*.^20^ Subsequently, a study was undertaken to isolate the antifungal metabolites. Phenazine and mesaconic acid were identified for the first time as the primary metabolites produced by the above-cited strain of *P. fluorescens* 9.^19^ Thus, phenazine, being a well-known antimicrobial metabolite, and its natural analogs phenazine-1-carboxylic acid (PCA) and 2-hydroxyphenazine (2-OH P), and some semisynthetic analogs as four mono- and dinitro derivatives were assayed against *M. phaseolina*, *Cercospora nicotianae* Ellis & Everhart, and *Colletotrichum truncatum* (Schweinitz) Andrus & W. D. Moore, the most dangerous fungal pathogens of soybean. Phenazine and PCA showed very strong inhibition against the three pathogens, while mesaconic acid and 2-OH P were practically inactive. The results of SAR studies demonstrated that the antifungal activity depends on the nature and the position of the substituent in the phenazine tricyclic system.^19^ In addition, the strain of *Pseudomonas donghuensis* SVBP6, exhibiting a broad antifungal activity, which was essentially due to 7-hydroxytropolone, was collected in Argentina. This compound, as well as its analogues, could be easily synthesized and bioformulated for potential practical application as a fungicide.^21^

Leaf blight of soybean is another severe disease of this crop induced by different species of *Cercospora*, which is one of the largest genera of hyphomycetes containing more than 650 species. *Cercospora kikuchii* (Matsumoto & Tomoyasu) Gardner is found worldwide, and *Cercospora nicotianae* has recently been recognized as a pathogen of soybean in Bolivia and Mexico.^22^

Anthracnose, caused by different *Colletotrichum* species, is another important factor limiting soybean production. The anthracnose losses are considered less severe than those caused by charcoal rot; however, they reduce the production of this legume by 50%. *C. truncatum* is the main causal agent of soybean anthracnose, which is characterized by pre- and postemergence damages on cotyledons, pods, petioles, and stems. On leaves, necrotic laminar veins can also be observed in premature defoliation. Symptoms may evolve into premature germination of grains, pod rot, and immature opening of pods.^23^

*Colletotrichum* spp. are able to synthesize a plethora of secondary metabolites belonging to diverse families of natural compounds and with interesting biological activities, including phytotoxins. Studies on the structural determination and biosynthesis of these metabolites were reviewed by García-Pajón and Collado.^24^

Among *Colletrichum* pathogenic species, some have been studied for the production of toxic metabolites such as *Colletotrichum gloeosporioides* (Penzig) Penzig & Saccardo, which is a widespread pathogen found on strawberries, grapes, etc.; *Colletotrichum nicotianae*, which is the causal agent of tobacco anthracnose disease; *Colletotrichum capsici* (Sydow) E.J. Butler & Bisby, which is a pathogen on peanuts, soybean, cowpea, etc.; and *Colletotrichum fragaria* A.N. Brooks and *Colletotrichum dematium* (Pers.) Grove, found on strawberries and beans.^24^ From *Colletotrichum higginsianum* Sacc. isolated in 1991 in Trinidad from diseased leaves of *Brassica rapa* subsp. *chinensis* (Linnaeus) Hanelt, some researchers isolated two specialized diterpenoid α-pyrones, named higginsianins A and B, which showed *in vitro* cytostatic activity.^25^ These preliminary activities were deeply investigated, and the results showed that higginsianins A and B can be considered promising anticancer agents due to their cytotoxic activities.^26^ From the same fungal cultures, a tetrasubstituted pyran-2-one and a tetrasubstituted dihydrobenzofuran, named colletochlorins E and F, respectively, were purified together with colletopyrone, colletochlorin A, and 4-chlororcinol. Higginsianins E and F showed antiproliferative activity against two human cancer cell lines (A431 and H1299) and were almost nontoxic against immortalized keratinocyte.^27^

Previously, *meso*-butane-2,3-diol, 2-hydroxymethylhexa-2,4-dienol, and colletruncoic acid methyl ester polyketide were isolated from a strain of *C. truncatum*, obtained from the American Type Culture Collection, Rockville, Md., as ATCC. However, no biological activity was reported for this fungus.^28^

Based on these literature data and considering that the same fungi could produce different metabolites if collected in different world regions or if grown *in vitro* in different media or conditions, a study was undertaken to investigate the bioactive metabolites synthesized by *C. truncatum* isolated from infected soybean collected in Argentina.

This article describes the purification and chemical and biological characterization of specialized bioactive disubstituted nonenolide and trisubstituted oct-2-en-4-one, named truncatenolide and truncatenone, respectively, tyrosol, and *N*-acetyltyramine, from a virulent strain of *C. truncatum* collected in Argentina from infected soybean plants. In particular, the antifungal activity of truncatenolide was also described in view of its potential as a biofungicide. Some close related fungal nonenolides were assayed in comparison to truncatenolide against *C. nicotianae* producing interesting results. Furthermore, the SAR results obtained from these assays were also discussed.

## Materials and Methods

### General Experimental Procedures

IR spectra were recorded on a PerkinElmer Spectrum 100 FT-IR spectrometer (Milan, Italy) as a glassy film; UV and ECD spectra were measured on a JASCO (Tokyo, Japan) J1500 spectropolarimeter at room temperature, using 0.5 mm cells at 4.7 mM in acetonitrile. ^1^H and ^13^C NMR spectra were taken at 400/100 MHz on a Bruker (Karleshrue, Germany) spectrometer in CDCl_3_ (used also as an internal standard). Bruker microprograms were used to perform COSY-45, HSQC, and HMBC experiments.^29^ HRESI and ESI mass spectra were recorded on a LC/MS TOF apparatus Agilent 6230B (Agilent Technologies, Milan, Italy). TLC (analytical and preparative) was carried out on SiO_2_ (Merck, Kieselgel 60 F254, 0.50 and 0.25 mm, respectively) plates (Merck, Darmstadt, Germany). Column chromatography was run on SiO_2_ (Merck, Kieselgel 60, 0.063–0.200 mm). UV light and/or spraying with 10% H_2_SO_4_ in MeOH and with 5% phosphomolybdic acid in EtOH, followed by heating at 110 °C for 10 min, were used to visualize the spots. All of the reagents and the solvents were purchased from Sigma-Aldrich Co. (Milan, Italy). Pinolidoxin and *epi*-pinolidoxin were obtained from *Dimydella pinodes* (syn. *Ascochyta punodes*) as previously reported,^30,31^ and 7,8-*O*,*O*′-diacetylpinolidoxin was obtained by usual acetylation of pinolidoxin.^30^ Stagonolide C^32^ and modiolide A and stagonolide H^33^ were obtained from *Stagonospora cirsii* as previously reported.

### Fungal Strain

The *C. truncatum* strain 17-5-5 was obtained from soybean having anthracnose symptoms in Roldàn, Sante Fe, Argentina, in 2017. The strain was deposited in the collection of the Plant Pathology Department of the University of Buenos Aires (FAUBA, Argentina). *M. phaseolina* strain 2013-1 and *C. nicotianae* strain Ck-2017-B34 used in the bioassays were deposited in the same collection.

### Production, Extraction, and Purification of the Metabolites

*C. truncatum* was grown on 4 L of potato dextrose broth (PDB) (DIFCO) constituted by potato starch (4.0 g/L) and dextrose (20.0 g/L) at 25 °C in the dark with shaking at 150 rpm for 18 days. The mycelium was separated by centrifugation (7000 rpm for 30 min), and the supernatant was filtered on 0.22 μm membranes (Whatman, Maidstone, UK) and lyophilized. The latter was redissolved in 400 mL of MilliQ H_2_O (Merck) (pH 6) and extracted with EtOAc (3 × 300 mL). The organic extracts were combined, dried (Na_2_SO_4_), and evaporated under vacuum. The yellow residue (294.6 mg) obtained was fractionated by SiO_2_ column, using as eluent CHCl_3_/*i*PrOH (9:1, v/v), yielding 10 groups of homogeneous fractions (F1–F10). F1 (17.1 mg) was purified by TLC, eluted with *n*-hexane/EtOAc (1:1, v/v), and yielded a homogeneous oily metabolite named truncatenone (**2**, 3.5 mg, *R*_f_ of 0.60). F2 (59.1 mg) was purified by TLC, eluted with *n*-hexane/EtOAc (1:1, v/v), and afforded four fractions (F2.1–F2.4). F2.2 (23.2 mg) was purified by TLC, eluted with CHCl_3_/*i*PrOH (97:3, v/v), and yielded a homogeneous oily metabolite named truncatenolide (**1**, 12.4 mg, *R_f_* of 0.46). F3 (43.5 mg) was purified by TLC, eluted with petroleum ether/acetone (7:3, v/v), and yielded an amorphous solid identified as tyrosol (**3**, 19.1 mg, *R*_f_ of 0.43). F7 (11.3 mg) was further purified by TLC, eluted with CHCl_3_/*i*PrOH (9:1, v/v), and yielded an amorphous solid identified as *N*-acetyltyramine (**4**, 2.4 mg, *R*_f_ of 0.20).

#### Truncatenolide (**1**)

UV (CH_3_CN) λ_max_ (log ε) 185 (3.6), 252 (1.9) nm; IR ν_max_ 3320, 1720, 1617, 1267 cm^–1^; ^1^H and ^13^C NMR, Table 1; HRESIMS: *m*/*z* 351.2179 [2M – H_2_O + H]^+^ (calcd for C_20_H_31_O_5_, 351.2172), 167.1078 [M – H_2_O + H]^+^ (calcd for C_10_H_15_O_2_, 167.1072).

**Table 1:** ^1^H and ^13^C NMR Data of Truncatenolide (**1**)^a^ and Its Esters (**5** and **6**)^b^

**1**				**5**	**6**
no.	δ_C_^c^	δ_H_ (*J* in Hz)	HMBC	δ_H_ (*J* in Hz)	δ_H_ (*J* in Hz)
2	172.7 s		H_2_C-3, H_2_C-4, H-10		
3	37.3 t	2.42 (1H) m^d^	H_2_C-4	2.43 (1H) m^d^	2.45 (1H) m^d^
		2.24 (1H) m^d^		2.25 (1H) m^d^	2.26 (1H) m^d^
4	30.2 t	2.42 (1H) m^d^	H-5, H-6, H_2_C-3	2.43 (1H) m^d^	2.45 (1H) m^d^
		2.24 (1H) m^d^		2.25 (1H) m^d^	2.26 (1H) m^d^
5	130.9 d	5.64 (1H) m	H_2_C-3, H_2_C-4	5.72 (1H) m	5.84 (1H) m
6	135.0 d	5.31 (1H) dd (15.4, 8.8)	H_2_C-4, H_2_C-8	5.29 (1H) dd (15.4, 8.8)	5.43 (1H) m^d^
7	74.9 d	4.11 td (8.8, 6.6)	H_2_C-8, H_2_C-9, H-5	5.17 td (8.8, 6.6)	5.43 (1H) m^d^
8	37.3 t	1.96 (1H) m	H-10, H_2_C-9	1.95 (1H) m	2.02 (1H) m
		1.61 (1H) m^d^		1.56 (1H) m	1.58 (1H) m
9	31.7 t	1.76 (1H) dd (14.3, 7.7)	CH_3_	1.79 (1H) m	1.90 (1H) m
		1.61 (1H) m^d^		1.61 (1H) m	1.70 (1H) m
10	72.8 d	4.89 (1H) br quint (6.5)	H_2_C-8, H_2_C-9, CH_3_	4.89 (1H) br quint (6.5)	4.95 (1H) br quint (6.5)
CH_3_	22.2 q	1.17 (3H) d (6.5)	H-9A	1.18 (3H) d (6.5)	1.20 (3H) d (6.5)
OAc				2.01 (3H) s	
2′–6′					7.86 (2H) d (8.3)
3′–5′					7.56 (2H) d (8.3)

a 2D ^1^H, ^1^H (COSY), and ^13^C, ^1^H (HSQC) NMR experiments confirmed the correlations of all of the protons and the corresponding carbons.

b Coupling constants (*J*) are given in parentheses.

c Multiplicities were assigned to the DEPT spectrum.

d These two signals are in part overlapped.

#### Truncatenone (**2**)

UV (CH_3_CN) λ_max_ nm (log ε) 196 (3.5), 273 (2.6) nm; IR ν_max_ 3011, 1718, 1635 cm^–1^; ^1^H and ^13^C NMR see Table 2; HRESIMS: *m*/*z* 171.1378 [M + H]^+^ (calcd for C_10_H_19_O_2_, 171.1385).

**Table 2:** ^1^H and ^13^C NMR Data of Truncatenone^a^^b^

no.	δ_C_^c^	δ_H_ (*J* in Hz)	HMBC
1	14.8 q	1.89 (3H) d (6.8)	H-2
2	138.3 d	6.79 (1H) q (6.8)	H_3_C-1, H_3_C-9
3	138.2 s		H_3_C-1, H_3_C-9
4	207.2 s		H-2, H-5, H-6, H_3_C-9, H_3_C-10
5	43.1 d	3.30 (1H) quint (7.2)	H_2_C-7, H_3_C-10
6	75.6 d	3.63 (1H) m	H-5, H_2_C-7, H_3_C-8, H_3_C-10
7	27.9 t	1.50 (1H) m	H_3_C-8
		1.43 (1H) m	
8	10.1 q	0.97 (3H) t (7.4)	H-6, H_2_C-7
9	10.8 q	1.78 (3H) s	H-2
10	16.0 q	1.15 (3H) d (7.2)	H-5

a 2D ^1^H, ^1^H (COSY), and ^13^C, ^1^H (HSQC) NMR experiments confirmed the correlations of all of the protons and the corresponding carbons.

b Coupling constants (*J*) are given in parentheses.

c Multiplicities were assigned to the DEPT spectrum.

#### Tyrosol (**3**)

^1^H NMR (CD_3_OD) δ 7.20 (d, *J* = 8.0 Hz, H-2, H-6), 6.80 (d, *J* = 8.0 Hz, H-3, H-5), 3.80 (t, *J* = 6.4 Hz, H_2_-8), 2.80 (t, *J* = 6.4 Hz, H_2_-7). ESI MS (+), *m*/*z*: 299 [2M + Na]^+^, 139 [M + Na]^+^.

#### *N*-Acetyltyramine (**4**)

^1^H NMR (CD_3_OD) δ 7.00 (d, *J* = 8.4 Hz, H-2, H-6), 6.70 (d, *J* = 8.4 Hz, H-3, H-5), 3.31 (t, *J* = 7.3 Hz, H_2_-8), 2.67 (t, *J* = 7.3 Hz, H_2_-7), 1.89 (s, Me-10). ESI MS (+), *m/z*: 180 [M + H]^+^.

#### 7-O-Acetyltruncatenolide (**5**)

To truncatenolide (**1**, 1.0 mg), dissolved in pyridine (10 μL), Ac_2_O (10 μL) was added. The reaction was performed overnight at room temperature and was stopped by MeOH addition. The azeotrope formed by benzene addition was evaporated under a N_2_ stream. The residue (1.2 mg) was purified by analytical TLC, using petroleum ether/acetone (95:5, v/v) as eluent, affording 7-*O*-acetyl truncatenolide (**5**, 1.1 mg). ^1^H NMR see Table 1; ESIMS (+) *m*/*z*: 249 [M + Na]^+^.

#### 7-O-*p*-Bromobenzoyltruncatenolide (**6**)

To truncatenolide **1** (1.7 mg) in CH_3_CN (100 μL), were added 4-dimethylaminopyridine (DMAP) (5 mg) and *p*-bromobenzoyl chloride (5 mg). The reaction was carried out for 4 h under stirring at room temperature and then dried. The residue (2.7 mg) was purified by analytical TLC, using as eluent CHCl_3_/*i*PrOH (98:2, v/v), giving derivative **6** (2.0 mg). ^1^H NMR see Table 1. ESIMS (+) *m*/*z*: 369 [M + 2 + H]^+^ and 367 [M + H]^+^.

### Computational Section

Molecular mechanics and density functional theory (DFT) calculations were run with Spartan’20 (Wavefunction, Inc., Irvine CA, 2021), with standard parameters and convergence criteria. Time-dependent DFT (TD-DFT) calculations were run with Gaussian’16 with default grids and convergence criteria.^34^ Two isomers each for compounds **1** and **2** were taken into consideration, namely, (7*R*,10*R*)-**1** and (7*S*,10*R*)-**1** and (5*S*,6*S*)-**2** and (5*R*,6*S*)-**2**. First, the conformational space of **1** and **2** was sampled with the Monte Carlo algorithm in Spartan'20 using Merck molecular force field (MMFF) by rotating all relevant single bonds (including endocyclic ones). All conformers thus found within an energy window of 10 kcal/mol (90 conformers for **1** and 87 for **2**) were first screened by single-point calculations at the B3LYP-D3/6-31G(d) level in vacuo, keeping the most stable and all those up 3.6 kcal/mol away from the most stable, then optimized at the B3LYP/6-31G(d) level in vacuo. Their populations were estimated at the B97M-V/6-311+G(2df,2p) level in vacuo (for NMR calculations). For ECD calculations, the set of conformers was reoptimized at the ωB97X-D/6-311G(d,p) level, including the SMD solvent model for acetonitrile. The procedure led to 4–6 conformers for **1** and 4–7 conformers for **2** and with a sizable population at 300 K. Relevant conformers for (7*R*,10*R*)-**1** are shown in the Supporting Information. Following the procedure established by Hehre et al. and implemented in Spartan’20,^35^ NMR GIAO calculations of ^13^C shieldings were run at the B3LYP/6-31G(d) level using structures optimized at the same level and Boltzmann averaged using populations estimated at the B97M-V/6-311+G(2df,2p) level. The DP4 test was run using the procedure implemented in Spartan’20, while the DP4+ test was run using scaled chemical shifts and the spreadsheet provided by Grimblat et al.;^36^ tables are available in the Supporting Information. Scalar H–H couplings were calculated at the B3LYP/PCJ-0 level using structures optimized at the B3LYP/6-31G(d) level and Boltzmann averaged using populations estimated at the B97M-V/6-311+G(2df,2p) level. All NMR calculations were run in vacuo. TD-DFT calculations were run as previously reported^17^ but using PCM solvent model for acetonitrile. The calculations included 36 excited states (roots). ECD spectra were generated as previously reported.^17^ The calculated spectra in Figure 2 were plotted with SpecDis v1.71 (https://specdis-software.jimdo.com/); they are red-shifted by 5 nm and scaled by a factor of 3 to compare with the experimental spectra.

### Soybean Seedling Bioassay

Compounds **1**–**4**, first dissolved in 5% of MeOH, were brought up to 2.5 × 10^–3^ mol/L with MilliQ H_2_O. Then, 1 mL of the solution of each sample and concentration was pipetted onto the surface of filter papers contained in three 6 cm Petri dishes. Seeds treated with 5% MeOH were used for the control treatment. Cytochalasin B, isolated from the fungus *Pyrenophora semeniperda* was used as a positive control at the same concentration.^37^ Four soybean seeds were placed onto the surface of each filter paper. The Petri dishes were incubated at 24 °C with a 16/8 light/dark photoperiod for 3 days. The seedling coleoptile and radicle length were measured using electronic calipers for 3 days. The experiment was repeated in triplicate with three independent trials.

### Antifungal Bioassay

The antifungal activity potential of compounds **1**–**4** was assayed against *M. phaseolina*.^17^ Briefly, *M. phaseolina* mycelial plugs (4-day-old culture) of 5 mm diameter were located in the center of potato dextrose agar (PDA) plates. For each compound, amounts of 2.5 × 10^–3^ mol/L were dissolved in 20 μL of 5% MeOH and applied to the tops of the mycelial plugs. 5% MeOH alone was used as a negative control and applied to the fungal plug. The solvent was allowed to evaporate in a laminar flow cabinet, and the plates were incubated at 28 °C for 5 days. The same procedure was performed to test compounds **7–12** against *C. nicotianae* and *M. phaseolina.* The percentage of inhibition of the fungal growth was calculated using the following formula
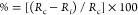
where *R*_c_ is the radial growth of the test pathogen in the control plates (mm), and *R_i_* is the radial growth of the test pathogen in the presence of compounds tested (mm). The experiment was repeated thrice.

### Statistical Analysis

GraphPad Prism 8 software was used to perform all of the statistical analyses. Data were expressed as mean ± SEM. Differences among groups were compared by one-way ANOVA. Differences were considered statistically significant at *p* < 0.05.

## Results and Discussion

**Figure 1:**
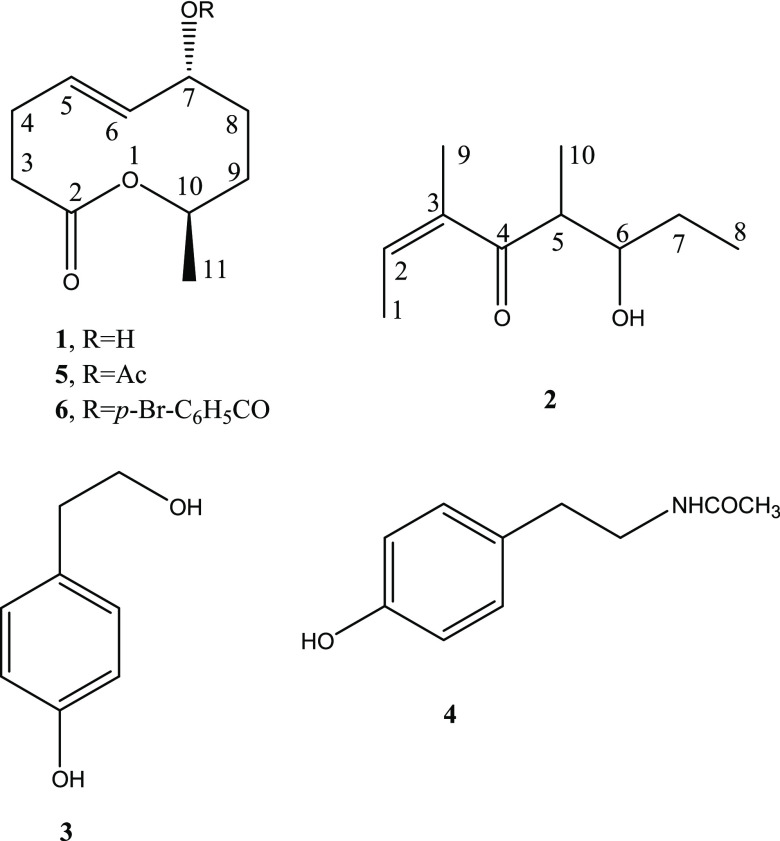
Structures of truncatenolide, truncatenone, tyrosol, and acetyltyramine (**1**–**4**) and those of 7-*O*-acetyl- and 7-*O*-*p*-bromobenzoyl-truncatenolide (**5** and **6**).

Two previously undescribed metabolites, named truncatenolide and truncatenone (**1** and **2**, Figure 1), and two known compounds identified as tyrosol and acetyltyramine (**3** and **4**, Figure 1) were obtained from the culture filtrate organic extract of *C. truncatum*, as described in the Materials and Methods section.

Metabolites **3** and **4** were identified by comparison of their spectroscopic data with those reported in the literature for **3** by Kimura and Tamura,^38^ Capasso et al.,^39^ and Cimmino et al.^40^ and for **4** by Lin et al.^41^ Tyrosol, which is already reported as a phytotoxic metabolite, was previously isolated from the cultures of different plant pathogens, i.e., *Diplodia seriata*,^42^*Alternaria tagetica*,^43^*Neofusicoccum parvum*,^44^ some *Lasiodiplodia* spp.,^40^ and *Diaporthella cryptica*.^45^ Recently, compound **3** was also produced by an endophytic fungus collected in India from *Houttuynia cordata* Thunb. Tyrosol showed strong antimicrobial activity against a plethora of clinically severe pathogens such as *Staphylococcus aureus*, *Candida albicans*, *Pseudomonas aeruginosa*, and *Escherichia coli*.^46^ Acetyltyramine (*N*-(4-hydroxyphenetyl)acetamide) belongs to the alkylamide family of naturally occurring compounds found in at least 33 plant families. Considering their structural variability associated with important biological activities (such as antimicrobial, immunomodulatory, larvicidal, antiviral, antioxidant, and insecticidal properties), they were recently the object of an extensive review focused essentially on cinnamoyltyramine.^47^ In particular, *N*-acetyl- and *N*-propionyl-tyramine were isolated together with tyrosol, several cyclic dipeptides, nucleosides and their aglycones, and *N*-acetyltryptamine and pyrrole-2-carboxylic acid from an endophytic *Streptomyces* sp. (AC-2) which was obtained from the root of the parasitic plant *Cistanche deserticola*.^41^

From the same organic extract of *C. truncatum*, two specialized metabolites were isolated and named on the basis of their structural features as truncatenolide and truncatenone (**1** and **2**, Figure 1). From preliminary spectroscopic analysis they seemed to belong to different groups of natural products, although both probably originated as polyketides.^48^

Truncatenolide (**1**) has a molecular formula of C_10_H_16_O_3_ as obtained from its HR ESIMS spectrum and is consistent with the hydrogen deficiency index equal to 3. The first analysis of its ^1^H and ^13^C NMR spectra showed signals typical of an ester moiety, hydroxylated secondary carbons, and an olefinic group, which were consistent with the signals observed in the IR^49^ and UV spectra.^50^ Considering the presence of a carbonyl and double bond, the remaining unsaturation should be due to a lactone ring.

In addition, the investigation of ^1^H NMR and COSY spectra^29^ (Table 1) showed the presence of a multiplet (H-5) and a double doublet (H-6) (*J*= 15.4 and 8.8 Hz) at δ 5.64 and 5.31 due to the protons of a *trans*-disubstituted double bond. H-6 coupled with the proton (H-7) of the adjacent hydroxylated secondary carbon resonating as a double triplet (*J* = 8.8 and 6.6 Hz) being coupled also with the protons of the adjacent methylene group (H_2_C-8) appearing as two multiplets at δ 1.96 and 1.61. The latter coupled with the protons of another methylene group (H_2_C-9) observed as a double doublet (*J* = 14.3 and 7.7 Hz) and a multiplet at δ 1.76 and 1.61 being also coupled with the proton (H-10) of a methine resonating as a quintet (*J* = 6.5 Hz) at δ 4.89. This secondary oxygenated carbon (C-10) is likely the closure point of the lactone ring. H-10, in turn, coupled with the protons of a geminal methyl group (H_3_-11) resonating as a doublet (*J* = 6.5 Hz) at δ 1.17. The other olefinic proton (H-5) coupled with the protons of the adjacent methylene group (H_2_C-4), which was observed as two multiplets at δ 2.42 and 2.24, were overlapped with the signals of the protons of the other methylene group (H_2_-3) α-located to the ester carbonyl group.^50^ The protonated carbons were assigned on the basis of the correlations observed in the HSQC spectrum.^29^ Thus, the signals at δ 135.0, 130.9, 74.9, 72.8, 37.3 (two overlapped signals), 31.7, 30.2, and 22.2 were assigned to C-6, C-5, C-7, C-10, C-3 and C-8, C-9, C-4, and C-11, respectively. The remaining singlet at the typical chemical shift value of δ 172.7 was assigned to the ester carbonyl group (C-2).^51^ Thus, the chemical shifts were assigned to all of the carbons, and corresponding protons of **1** and truncatenolide were formulated as 7-hydroxy-10-methyl-3,4,7,8,9,10-hexahydro-2*H*-oxecin-2-one (**1**).

This structure was supported by the long-range couplings observed in the HMBC spectrum^29^ (Table 1). Significant were the couplings observed between C-2 and H_2_C-3, H_2_C-4 and H-10; C-5 with H_2_C-3 and H_2_C-4; C-6 with H_2_C-4 and H_2_C-8; and C-10 with H_2_C-8, H_2_C-9, and H_3_C-11. The HR ESI MS spectrum showed both the ions produced by loss of H_2_O from the protonated dimer [2M – H_2_O + H]^+^ and from the protonated adduct [M + H – H_2_O]^+^ at *m*/*z* 351.2179 and 167.1078, respectively.

The structure of truncatenolide was confirmed by preparing two key ester derivatives (**5** and **6**) by acetylation and *p*-bromobenzoylation of the C-7 hydroxy group. The ^1^H NMR spectrum of derivative **5** (Table 1) differed from that of **1**, recorded in the same conditions, for the typical downfield shift of H-7 (Δδ 1.06) appearing as a triple doublet (*J* = 8.8 and 6.6) at δ 5.17 and for the presence of the singlet of the acetyl group at δ 2.01. Its ESIMS spectrum showed the sodium [M + Na]^+^ adduct ion at *m*/*z* 249. The ^1^H NMR spectrum of derivative **6** (Table 1) differed from that of **1**, recorded in the same conditions, for the typical downfield shift of H-7 (Δδ 1.32) resonating as a multiplet at δ 5.43 being overlapped to the signal of H-6 and for the typical pattern system of the *p*-bromobenzoyl system appearing as two coupled doublets (*J* = 8.3 Hz) at δ 7.86 and 7.56. Its ESI MS spectrum showed the typical signals of the protonated adduct as a result of the presence of ^81^Br and ^79^Br isotopic peaks at *m*/*z* 369 [M + 2 + H]^+^ and 367 [M + H]^+^, respectively.

The *E* stereochemistry of the double bond was determined from the coupling between the two olefinic protons (*J* = 15.4 Hz).^50^ The relative configuration of the two stereogenic centers was obtained by DP4 and DP4+ analysis,^35,52^ based on NMR GIAO calculations of ^13^C shieldings run at the B3LYP/6-31G(d) level. Additionally, ^3^*J*_HH_ couplings were estimated both empirically (Karplus-type relation) from DFT structures and by calculating Fermi contact (FC) scalar couplings at the B3LYP/pcJ-0 level. For both kinds of calculations (shieldings and scalar couplings), a whole conformational set was employed (see the Computational Section), and the estimated or calculated values represent Boltzmann averages using populations evaluated at the B97M-V/6-311+G(2df,2p)//B3LYP/6-31G(d) level of theory. The DP4/DP4+ probability levels were 100% for the *rel*-(7*R*,10*R*) isomer of **1** and 0% for the *rel*-(7*S*,10*R*) one. The diagnostic ^3^*J*_H5,H6_ ≈ 9 Hz (measured) was reproduced for the *rel*-(7*R*,10*R*) isomer (empirical estimation from DFT structures, 11.5 Hz; FC calculations, 9.7 Hz) but not for the *rel*-(7*S*,10*R*) isomer (empirical, 4.6 Hz; FC, 3.0 Hz).

**Figure 2:**
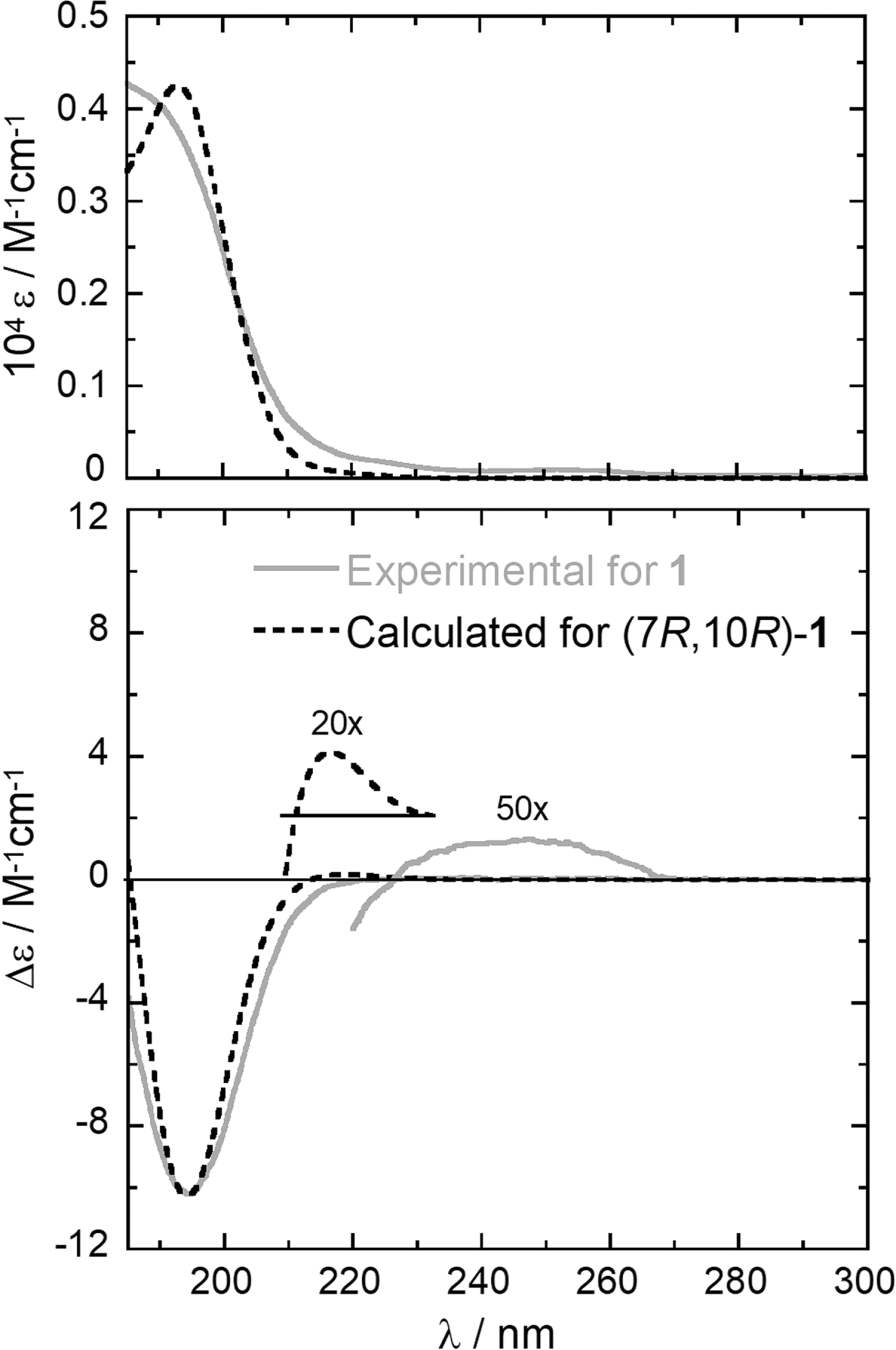
Comparison between experimental UV (top) and ECD spectra (bottom) measured for truncatenolide (**1**) and calculated at the CAM-B3LYP/def2-TZVP/PCM//ωB97X-D/6-311+G(d,p)/SMD level. See the Experimental and Computational Sections for details.

The ECD spectrum of truncatenolide (Figure 2) showed a major negative band centered at 195 nm, allied with the alkene π–π* transition, and a weak and broad positive band above 230 nm, mainly due to the ester n−π* transition. Therefore, the absolute configuration could be determined by time-dependent DFT (TD-DFT) calculations.^53−55^ Using structures optimized at the ωB97X-D/6-311+G(d,p) level, including SMD solvent model for acetonitrile, TD-DFT calculations were run at CAM-B3LYP/def2-TZVP and B3LYP/def2-TZVP levels, including the PCM solvent model for acetonitrile. CAM-B3LYP functional performed relatively better, as expected,^56^ and could reproduce not only the major ECD band but also the minor one (though blue-shifted with respect to the experiment, see Figure 2). In conclusion, the absolute configuration of truncatenolide (**1**) may be assigned as (7*R*,10*R*) and the compound determined as (*5E*,7*R*,10*R*)-7-hydroxy-10-methyl-3,4,7,8,9,10-hexahydro-2*H*-oxecin-2-one (**1**).

Nonenolides belong to the large family of macrolides which are polyketides biosynthesized from different organisms with a wide variety of biological activity, and thus they have been the object of several reviews.^57−60^ Several natural phytotoxic nonenolides are reported as fungal metabolites, such as pinolidoxins produced by *Didymella pinodes* (syn. *Ascochita pinodes*), the causal agent of pea anthracnose putaminoxin, herbarumins, and stagonolides produced by *Phoma putaminun*, *Phoma herbarum*, and *S. cirsii*, respectively; all fungi are proposed as potential mycoherbicides to control dangerous weeds such as *Erigeron annuus*, *Amaranthus retruflexus*, *Cirsium arvense*, and *Sonchus arvensisi*, infesting pastures and important agrarian cultures.^16,61,62^

Truncatenone (**2**, Figure 1) showed a molecular formula of C_10_H_18_O_2_ consistent with two hydrogen deficiencies. Its ^1^H and ^13^C NMR spectra (Table 2) showed the signals of an open chain hydroxylated enone in agreement with the bands observed for hydroxyl, unsaturated ketone, and double bond in its IR spectrum^49^ and the typical absorption maximum recorded in the UV spectrum.^50^ In particular, its ^1^H and COSY spectra (Table 1) showed the quartet (*J* = 6.8 Hz) of a proton (H-2) typical of a trisubstituted olefinic group at δ 6.79, which coupled with the germinal vinyl methyl (H_3_-1) appearing as a doublet (*J* = 6.8 Hz) at δ 1.89. The other vinyl methyl attached to the double bond resonated as a singlet at δ 1.78, and thus, the remaining bond of the olefinic group was with the carbonyl of the enone-constituting system observed in the ^13^C NMR spectrum (Table 1) at a typical chemical shift value of δ 207.2.^50,51^ The other substituent of the ketone group appeared to be the 1-methyl-2-hydroxybutyl residue. Its terminal methyl group (H_3_-8) resonated as a triplet (*J* = 7.4 Hz) at δ 0.97 being coupled with the protons (H_2_-7) of the adjacent methylene group, which appeared as two multiplets at δ 1.50 and 1.43 and also coupled with the multiplet of the proton (H-6) of the adjacent secondary hydroxylated carbon (C-6) at δ 3.63. This latter coupled with the adjacent methine proton (H-5) observed as a quintet (*J* = 7.2 Hz) at δ 3.30 being also coupled with the protons (H_3_-10) of a fourth methyl group resonating as a doublet (*J* = 7.2 Hz) at δ 1.15.^40^ The couplings observed in the HSQC spectrum (Table 2) allowed us to assign the protonated carbons present at δ 138.3, 75.6, 43.1, 27.9, 16.0, 14.8, 10.8, and 10.1 to C-2, C-6, C-5, C-7, C-10, C-1, C-9, and C-8, respectively. The olefinic tertiary carbon (C-3) was assigned at the singlet present at δ 138.2.^51^ The latter, as expected, in the HMBC spectrum (Table 2) showed significant long-range couplings with H_3_C-1 and H_3_C-9, as well as the carbonyl carbon (C-4) with the same protons. Thus, the chemical shifts of all of the carbons and corresponding protons of **2** were assigned as reported in Table 2, and truncatenone was formulated as 6-hydroxy-3,5-dimetyloct-2-en-4-one (**2**).

The structure assigned to truncatenone (**2**) was supported by the other couplings observed in the HMBC spectrum (Table 1) and by HR ESI MS data. The latter spectrum showed the protonated adduct ion [M + H]^+^ at *m*/*z* 171.1378.

The *Z* configuration of the double bond was determined by the NOESY spectrum.^29^ In fact, in addition to the expected correlation between H-2 and H_3_-1, a significant one between H-2 and H_3_-9 was also observed. The relative configuration of the two stereogenic centers was assigned in the same way as truncatenolide (**1**).^35,52^ The DP4/DP4+ probability levels were 99.9% for the *rel-*(5*S*,6*S*) isomer and 0.1% for the *rel-*(5*R*,6*S*) one. The assignment was further confirmed by the estimation of ^3^*J*_HH_ coupling constants.^63^ The observed diagnostic ^3^*J*_H5,H6_ ≈ 7 Hz was well reproduced for the *rel-*(5*S*,6*S*) isomer of **2** (empirical, 6.5 Hz; FC, 9.2 Hz) but not for the *rel*-(5*R*,6*S*) isomer (empirical, 1.3 Hz; FC, 1.2 Hz).

Truncatenone had a negligible electronic circular dichroism (ECD) spectrum above 190 nm and zero optical rotation. Thus, it was apparently isolated as a racemic mixture. We^64,65^ and others^66,67^ have previously isolated several racemic natural products, possibly originating from nonenzymatic pathways. Therefore, the final structure of truncatenone has to be indicated as *rel*-(5*S*,6*S*)-6-hydroxy-3,5-dimetyloct-2-en-4-one (**2**).

Truncatenone, to the best of our knowledge, is the first naturally occurring oct-2-en-4-one. Some cyclic compounds containing a similar moiety were found as synthetic and natural compounds. Among the last ones, there are cyclic monoterpenes such as verbenone, piperitone, and umbellulone.^48^

**Figure 3:**
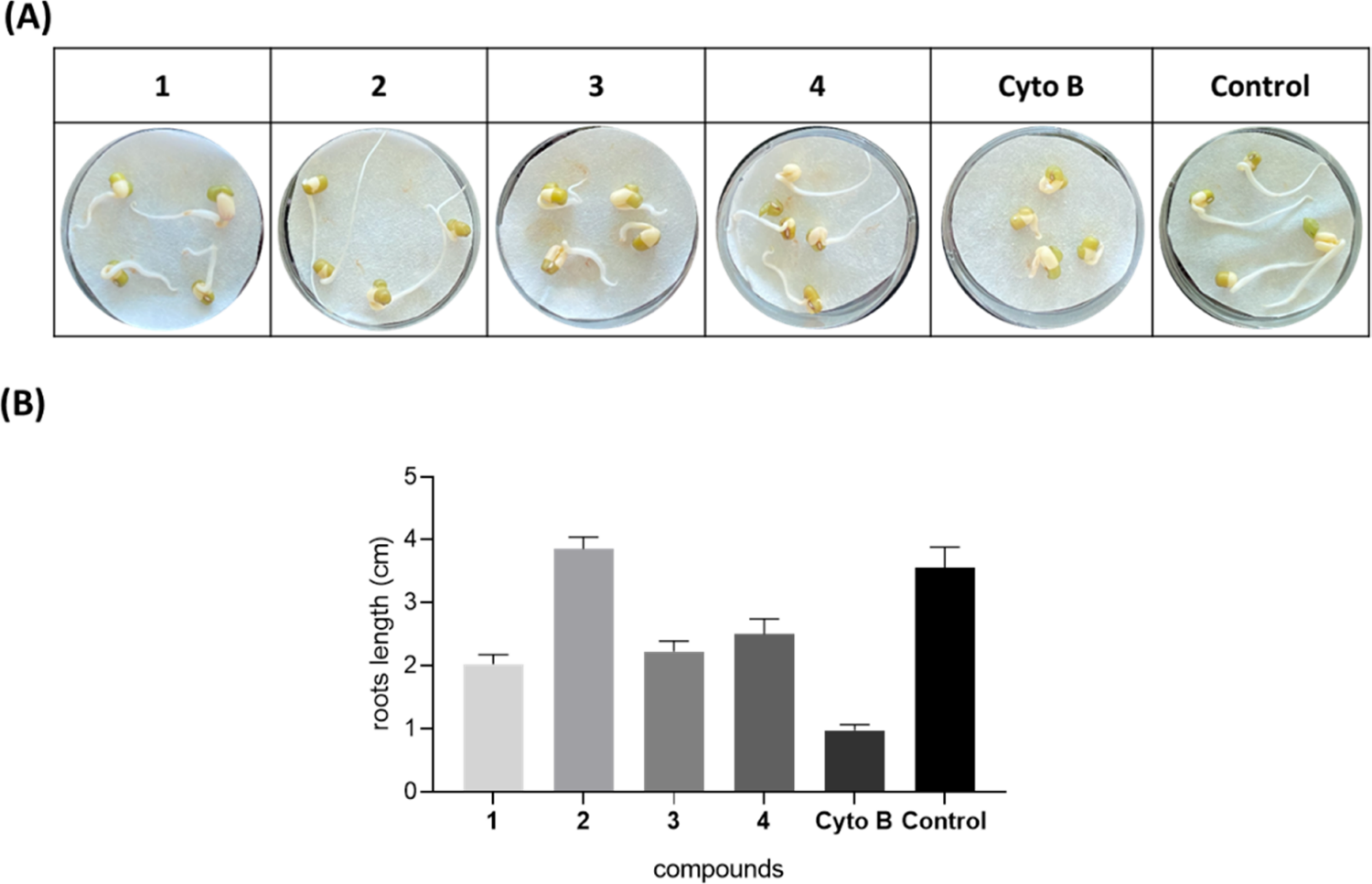
Germination test. (A) Representative photographs of seed viability. (B) Root length (cm) detection. **1**, truncatenolide; **2**, truncatenone; **3**, tyrosol; **4**, acetyltyramine; Cyto B, Cytochalasin B; and Control: 5% of MeOH. All compounds were tested at a final concentration of 2.5 × 10^–3^ mol/L. The experiment was performed in triplicate with three independent trials. Data are presented as means ± the standard deviation (*n* = 4) compared to the control. For comparative analysis of groups of data, one-way ANOVA was used, and *p* values < 0.0001 were extremely significant.

Compounds **1–4** were tested using a germination soybean seed bioassay at a final concentration of 2.5 × 10^–3^ mol/L as reported in the experimental section. Truncatenolide (**1**) shows phytotoxic activity with an evident inhibition of the growth of the soybean radicle of about 43% compared to the control (Figure 3). Tyrosol (**3**) and *N*-acetyltyramine (**4**) also showed phytotoxic activity, inhibiting the growth of the root of the seeds by 37 and 29%, respectively. Conversely, truncatenone (**2**) stimulated the growth of the seed root in comparison to the control by about 12%.

These results were also compared with the known phytotoxic metabolite cytochalasin B (Cyto B) isolated from *P. semeniperda*,^37^ used as a positive control to confirm the phytotoxicity. Cyto B shows strong phytotoxic activity inhibiting the growth of the soybean radicle by about 72%.

**Figure 4:**
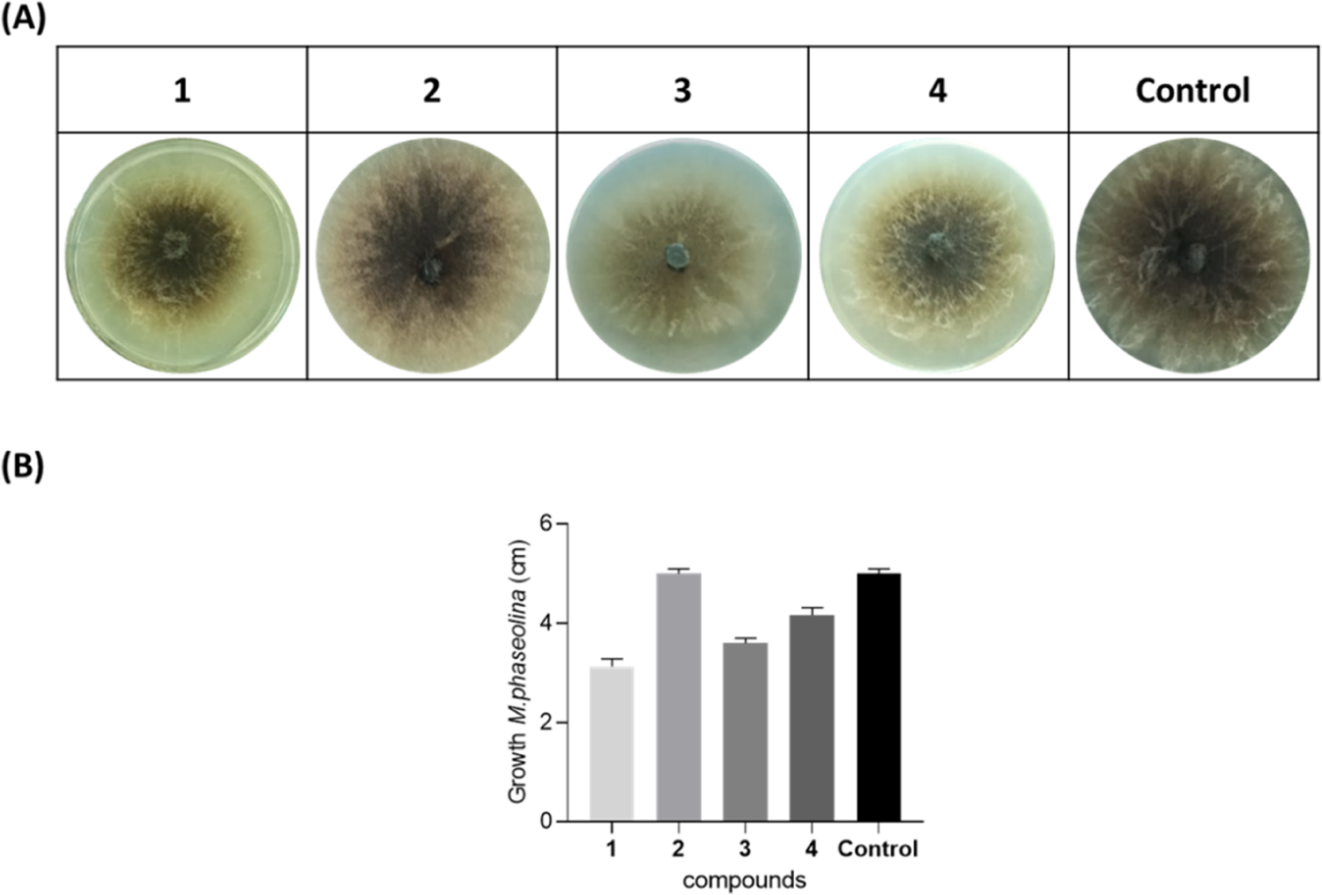
Antifungal Bioassay. (A) Representative photographs of the antifungal assay against *M. phaseolina*. (B) Detection of the inhibition of growth of *M. phaseolina* (cm). **1**, truncatenolide; **2**, truncatenone; **3**, tyrosol; **4**, acetyltyramine; and Control, *M. phaseolina*. All compounds were tested at a final concentration of 2.5 × 10^–3^ mol/L. The experiment was performed in triplicate with three independent trials. Data are presented as means ± the standard deviation (*n* = 4) compared to the control with a *p*-value < 0.001.

Compounds **1–4** were also tested against *M. phaseolina*, the main fungal pathogen of soybean and competitor of *C. truncatum*. As shown in Figure 4, truncatenolide (**1**) has the best antifungal activity compared to the other compounds tested, inhibiting fungal growth by around 40%. The compounds tyrosol (**3**) and *N*-acetyltyramine (**4**) show a slight antifungal activity of around 28 and 20%, respectively, and truncatenone (**2**) is shown to be inactive.

**Figure 5:**
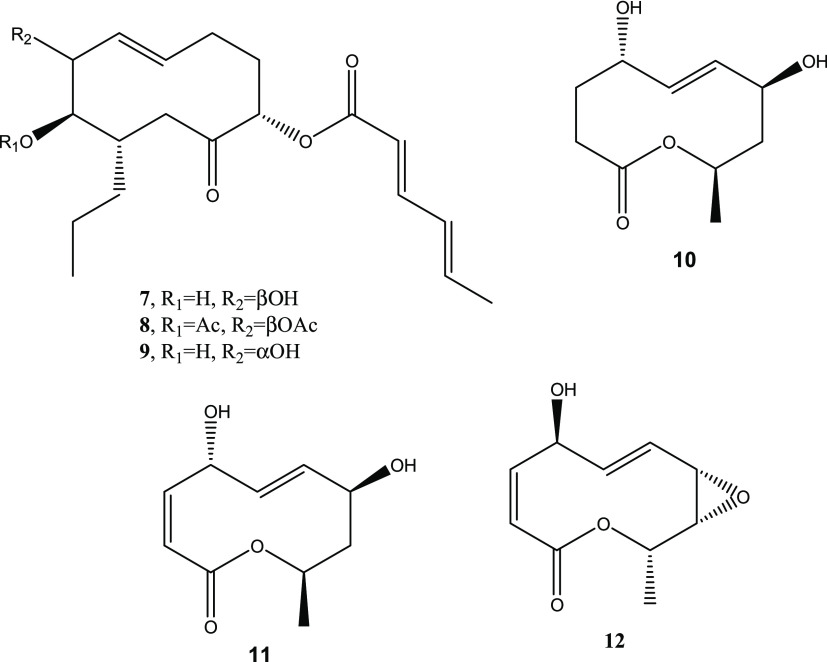
Structures of pinolidoxin and its 7,8-*O*,*O*′-diacetyl derivative (**7** and **8**), *epi*-pinolidoxin (**9**), stagonolides C and H (**10** and **12**, respectively), and modiolide A (**11**).

Considering the activity of truncatenolide and the availability of some close related nonenolides produced as bioactive metabolites from pathogenic fungi for agrarian plants^16^ and weeds,^61^ a structure–activity relationship study was performed. In this investigation, we used some bioactive fungal nonenolides, such as pinolidoxin and *epi*-pinolidoxin produced by *D. pinodes,*(30,31) the derivative 7,8-*O*,*O*′-diacetylpinolidoxin^31^ (**7**, **9**, and **8**, Figure 5), and stagonolide C^32^ and modiolide A and stagonolide H^33^ (**10**–**12**, Figure 5) obtained from *S. cirsii*. Their antifungal activity was tested against *C. nicotianae*, the causal agent of soybean anthracnose, and in comparison to truncatenolide (**1**).

**Figure 6:**
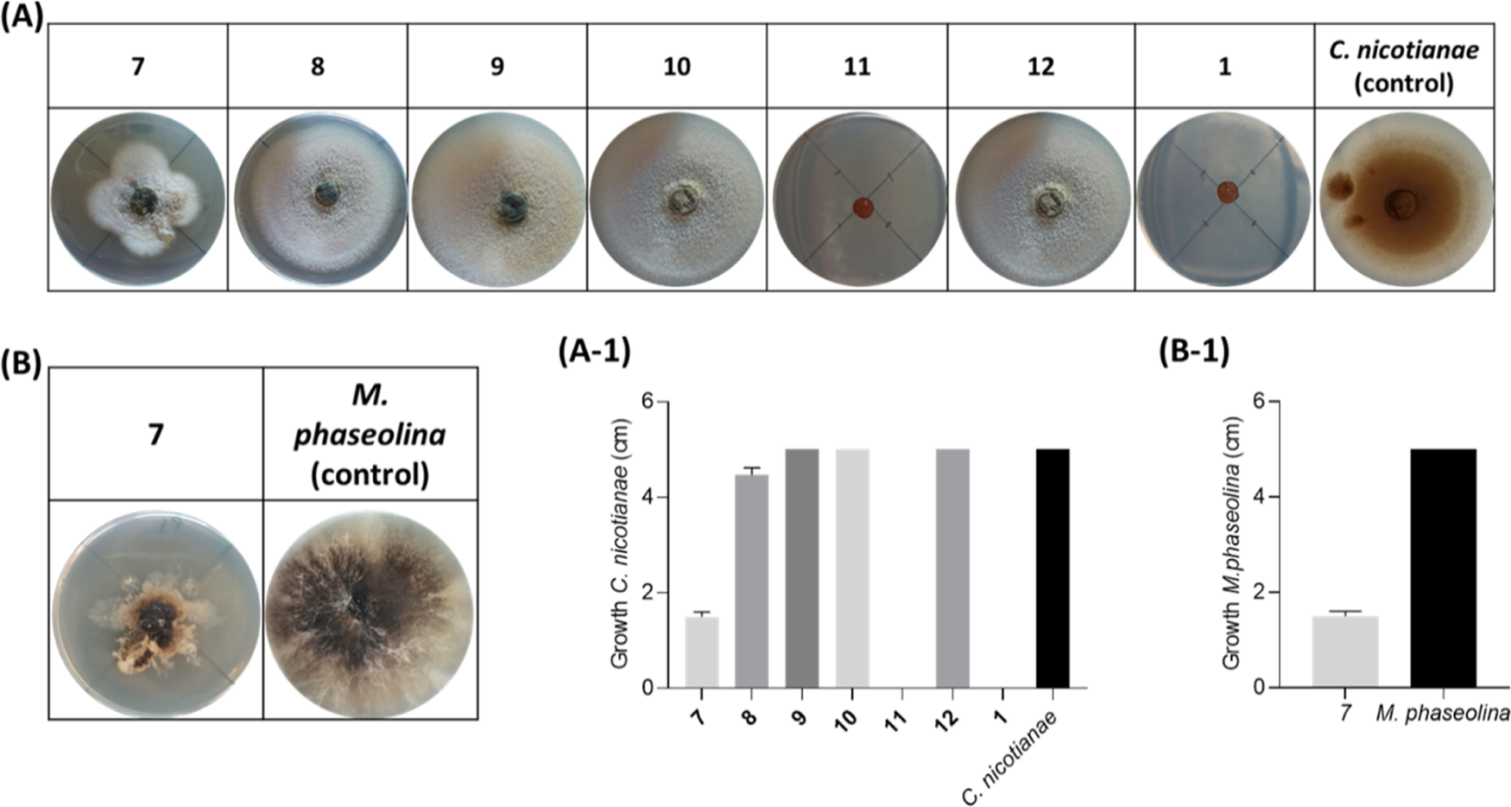
Antifungal Bioassay. (A) and (B) Representative photographs of the antifungal assay against *C. nicotianae* and *M. phaseolina*, respectively. Detection of the inhibition of growth of *C. nicotianae* (cm) and *M. phaseolina* (cm) (A-1 and B-1, respectively). **7**, pinolidoxin; **8**, *O*,*O*′-diacetylderivate; **9**, *epi*-pinolidoxin; **10**, stagonolide C; **11**, modiolide A; **12**, stagonolide H; and **1**, truncatenolide. All compounds were tested at a final concentration of 2.5 × 10^–3^ mol/L. The experiment was performed in triplicate with three independent trials. Data are presented as means ± the standard deviation (*n* = 4) compared to the control with a *p*-value < 0.001.

The results obtained by antifungal assay against *C. nicotianae* (Figure 6) surprisingly showed the strongest antifungal activity of truncatenolide (**1**), which was able to inhibit the fungal growth by 100%. The same result was obtained with modiolide A (**11**) and lower activity with pinolidoxin (**7**), which was able to inhibit the fungal growth by 75% (Figure 6, A-1). The compounds **8–10** and **12** were found to be inactive. When the same nonenolides (**7**–**12**) were tested against *M. phaseolina* in comparison to **1**, only pinolidoxin (**7**) inhibited the growth of the fungus by 75% (Figure 6, B-1). Thus, modiolide A (**11**) showed selective and strong growth inhibition of *C. nicotianae*.

These results did not surprise and are in agreement with those previously obtained by testing some of the known nonenolides above cited against other agrarian and weedy plants.^16,61^ The strong antifungal activity of truncatenolide against two fungi competitors such as *M. phaseolina* and *C. nicotianae* pathogens of soybean is very interesting as only a few other cases have been previously reported for different fungi pathogens of forest plants.^68^

The results of the SAR study testing nonenolides (**7**–**12**) against *C. nicotianae* in comparison to truncatenolide (**1**) showed that the integrity and the functionalities of the nonenolide ring are important for the antifungal activity. In particular, the nature of the substituent at the hydroxylated carbon involved in the lactone group did not affect this activity being *n*-propyl in **7** and methyl in **11**, as well as the hydroxylation of the carbon α-located to the carbonyl of the lactone group and its derivatization present only in **7** but not in **11**. The inactivity of the diacetyl derivative of **7** (compound **8**) could be due to the inefficacy of *C. nicotianae* to hydrolyze, in physiological conditions, the ester acetyl group at C-7 and C-8, converting it into pinolidoxin (**7**). Finally, the lack of toxicity of **9**, **10**, and **12** is not easy to be evaluated. However, as **9** differs from **7** for the epimerization of the hydroxy group at C-4, it can be deduced that to impart activity to this carbon if a hydroxy group is present, it could have β-configuration. Compound **12** differs from **11** only for the epoxy group, which could reduce the conformational freedom of the nonenolide ring and/or its recognition due to increased hindrance. These results are in agreement with those previously reported for some of the same cited nonenolides and other ones used in other SAR studies.^16,61^

In conclusion, a specialized and a disubstituted nonenolide, named truncatenolide, and a trisubstituted oct-2-en-4-one truncatenone (**1** and **2**) were isolated from the culture filtrates of *C. truncatum* pathogen of soybean in Argentina. Truncatenolide (**1**) was observed to be phytotoxic, inhibiting the growth of the soybean radicle in a germination soybean seed bioassay, and showed antifungal activity against *M. phaseolina*, while truncatenone (**2**) stimulated the growth of the seed root in comparison to the control. The antifungal activity of truncatenolide (**1**), which showed total growth inhibition of *C. nicotianae*, another fungal competitor responsible for different, but however, severe diseases of soybean, is noteworthy. The total inhibition and significant antifungal activity showed against *C. nicotianae* by modiolide A and pinolidoxin (**11** and **7**) is also interesting. Pinolidoxin (**7**) also showed similar activity against *M. phaseolina*. Thus, modiolide A had strong and selective activity against *C. nicotianae*. Truncatenolide, modiolide A, and to a lesser extent pinolidoxin (**1**, **11**, and **7**) showed potential antifungal activity, although other extensive and deep studies are needed to further evaluate their potential fungicidal activity.

## Supporting Information Available

^1^H NMR spectrum of truncatenone, **1** (S1); COSY spectrum of truncatenone, **1** (S2); NOESY spectrum of truncatenone, **1** (S3); HSQC spectrum of truncatenone, **1** (S4); HMBC spectrum of truncatenone, **1** (S5); ^13^C NMR spectrum of truncatenone, **1** (S6); HRESI MS spectrum of truncatenone, **1** (S7); ^1^H NMR spectrum of truncatenolide, **2** (S8); COSY spectrum of truncatenolide, **2** (S9); NOESY spectrum of truncatenolide, **2** (S10); HSQC spectrum of truncatenolide, **2** (S11); HMBC spectrum of truncatenolide, **2** (S12); ^13^C NMR spectrum of truncatenolide, **2** (S13); HRESI MS spectrum of truncatenolide, **2** (S14); conformers of truncatenolide (S15); and computational data (PDF)
