# Supplementary material for: Truncatenolide, a Bioactive Disubstituted Nonenolide Produced by Colletotrichum truncatum, the Causal Agent of Anthracnose of Soybean in Argentina: Fungal Antagonism and SAR Studies
Source: J Agric Food Chem. 2022 Aug 4;70(32):9834–44. doi: 10.1021/acs.jafc.2c02502 (PMC9389607; doi:10.1021/acs.jafc.2c02502)
Supplement: Supplementary file 1 — jf2c02502_si_001.pdf [file jf2c02502_si_001.pdf]

SUPPORTING INFORMATION

Truncatenolide a Bioactive Disubstituted Nonenolide Produced by *Colletotrichum truncatum*, the Causal Agent of Anthracnose of Soybean in Argentina. Fungal Antagonism and SAR Studies

Marco Masi, Stefany Castaldi, Francisco Sautua, Gennaro Pescitelli,* Marcelo Anibal Carmona and Antonio Evidente*

Supporting Information List

Page 3: **Figure S1**. ^{1}H NMR spectrum of trucantenolide, **1** ($CDCl_3$, 400 MHz).

Page 3: **Figure S2**. COSY spectrum of trucantenolide, **1** ($CDCl_3$, 400 MHz).

Page 4: **Figure S3**. NOESY spectrum of trucantenolide, **1** ($CDCl_3$, 400 MHz).

Page 4: **Figure S4**. HSQC spectrum of of trucantenolide, **1** ($CDCl_3$, 400/100 MHz).

Page 5: **Figure S5**. HMBC spectrum of of trucantenolide, **1** ($CDCl_3$, 400/100 MHz).

Page 5: **Figure S6.** ^{13}C NMR spectrum of trucantenolide, **1** ($CDCl_3$, 100 MHz).

Page 6: **Figure S7**. HRESI MS spectrum of of trucantenolide, **1** recorded in positive modality.

Page 6: **Figure S8**. ^{1}H NMR spectrum of truncatenone, **2** ($CDCl_3$, 400 MHz).

Page 7: **Figure S9**. COSY spectrum of truncatenone, **2** ($CDCl_3$, 400 MHz).

Page 7: **Figure S10**. NOESY spectrum of truncatenone, **2** ($CDCl_3$, 400 MHz).

Page 8: **Figure S11**. HSQC spectrum of truncatenone, **2** ($CDCl_3$, 400/100 MHz).

Page 8: **Figure S12**. HMBC spectrum of truncatenone, **2** ($CDCl_3$, 400/100 MHz).

Page 9: **Figure S13**. ^{13}C NMR spectrum of truncatenone, **2** ($CDCl_3$, 100 MHz).

Page 9: **Figure S14**. HRESI MS spectrum truncatenone, **2** recorded in positive modality.

Page 10: **Figure S15.** Conformers of truncatenolide, **1**

Page 11: **Table S1** and **Table S2.** Computational data (DP4 analysis).

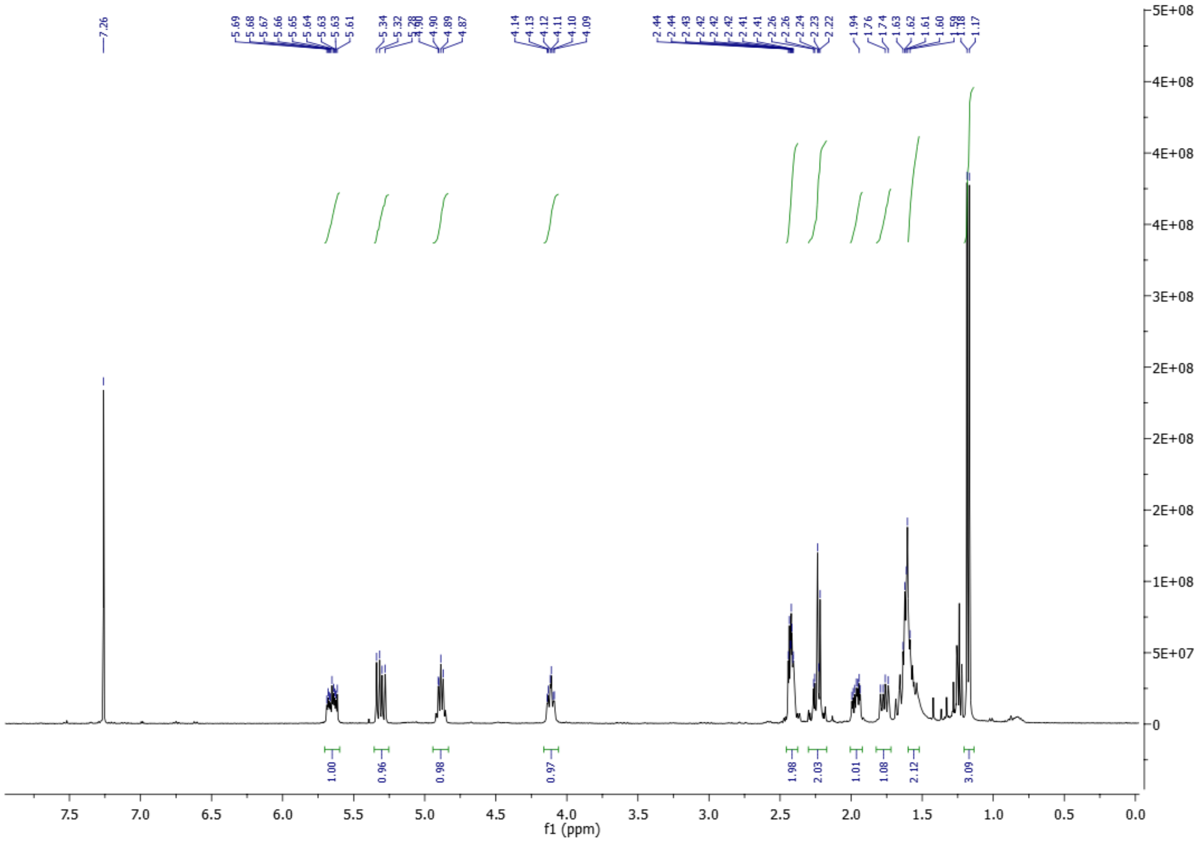

Figure S1. ^{1}H NMR spectrum of trucantenolide, **1** ($CDCl_3$, 400 MHz).

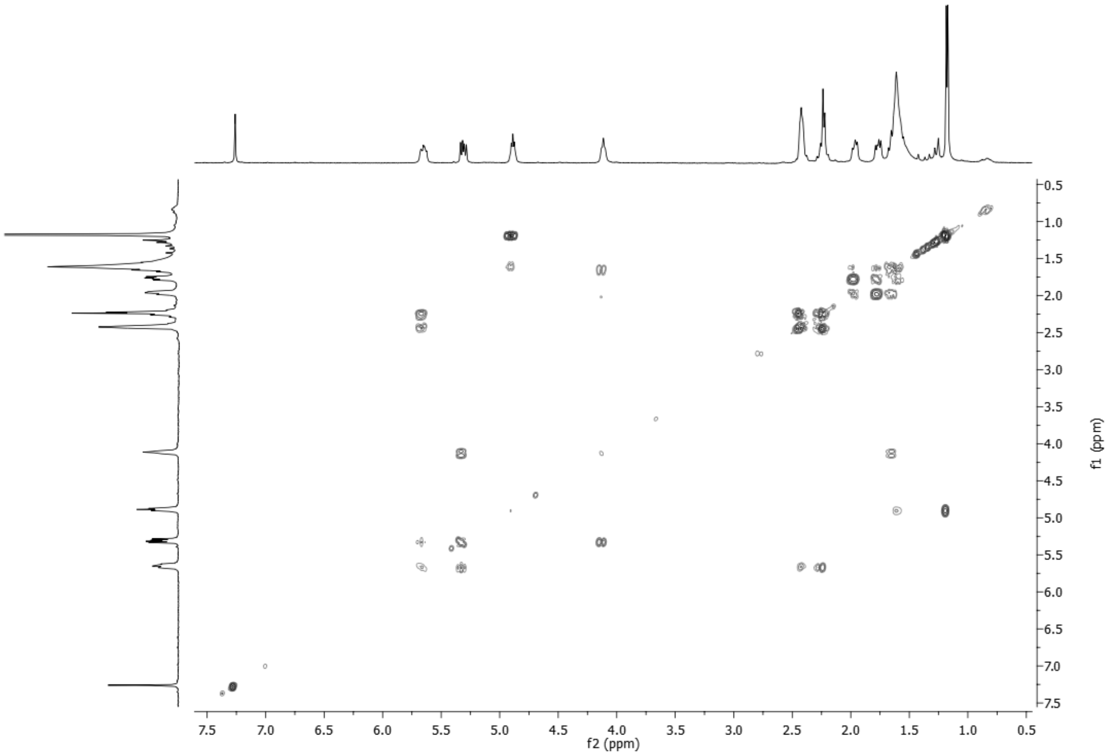

Figure S2. COSY spectrum of trucantenolide, **1** ($CDCl_3$, 400 MHz).

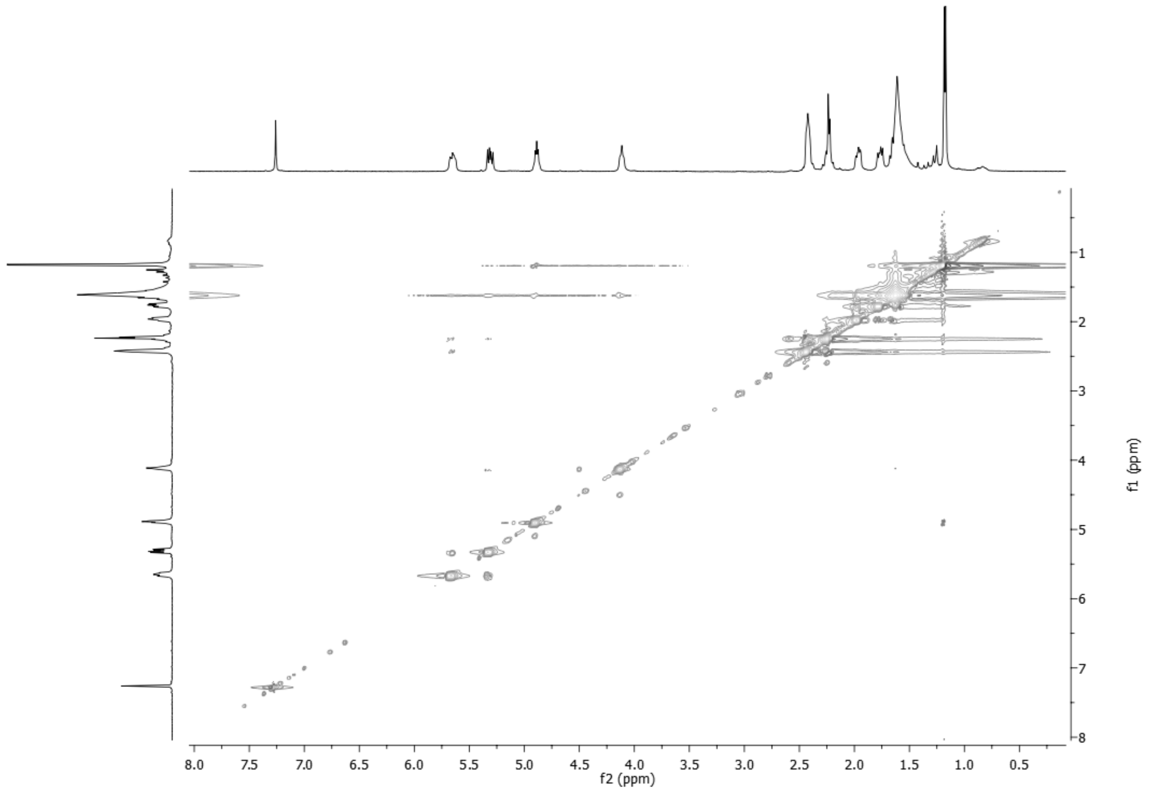

Figure S3. NOESY spectrum of trucantenolide, **1** ($CDCl_3$, 400 MHz).

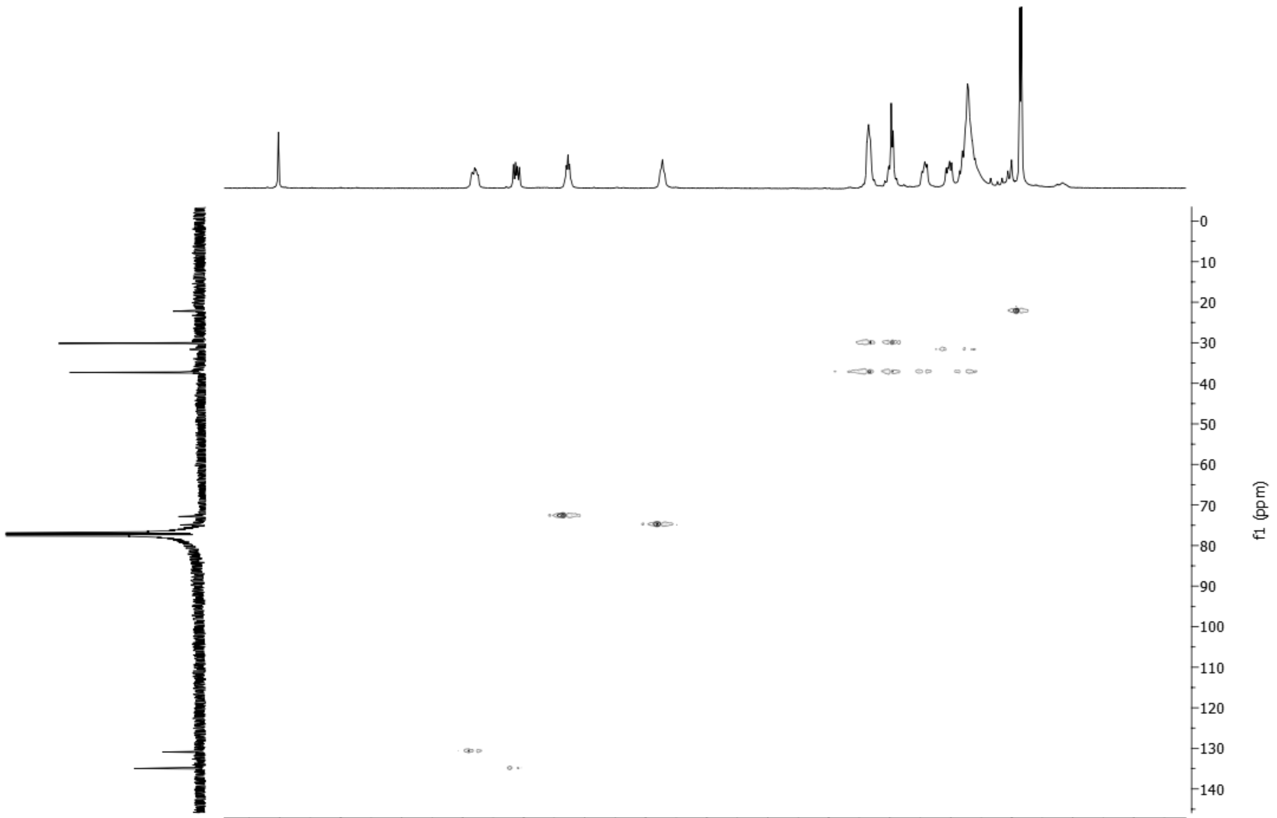

Figure S4. HSQC spectrum of of trucantenolide, **1** ($CDCl_3$, 400/100 MHz).

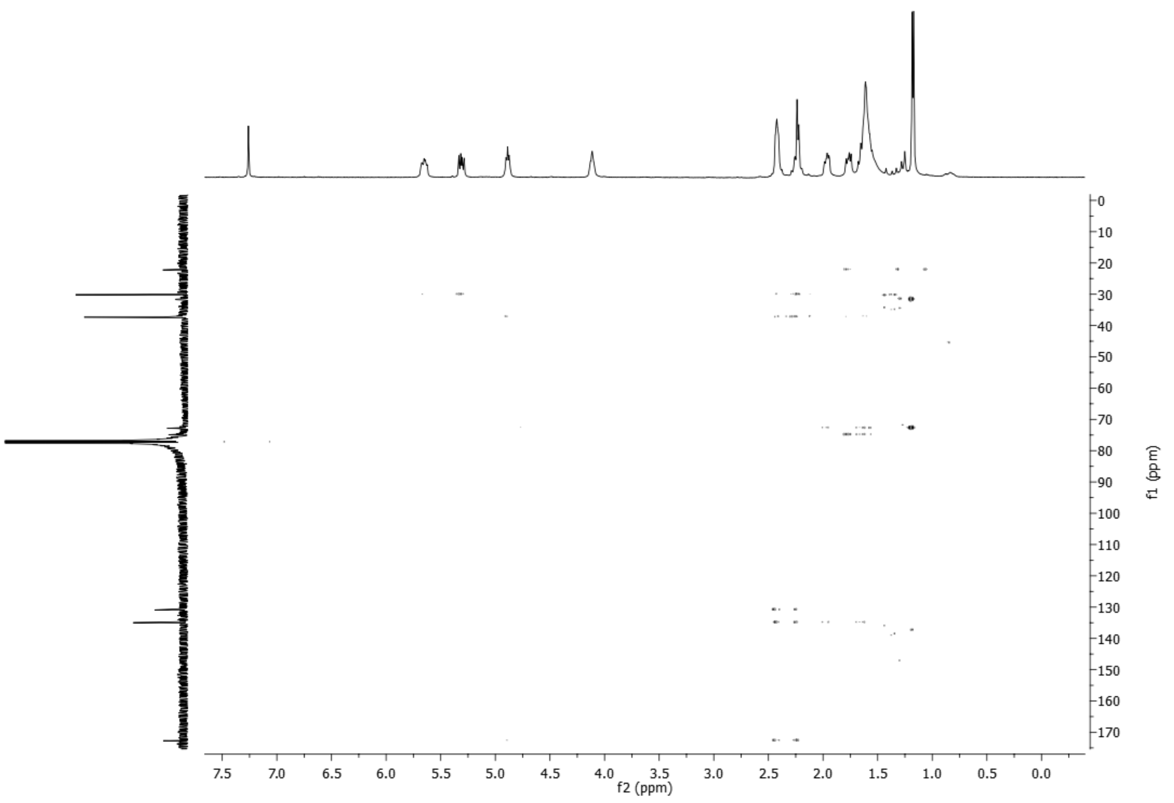

Figure S5. HMBC spectrum of of trucantenolide, **1** ($CDCl_3$, 400/100 MHz).

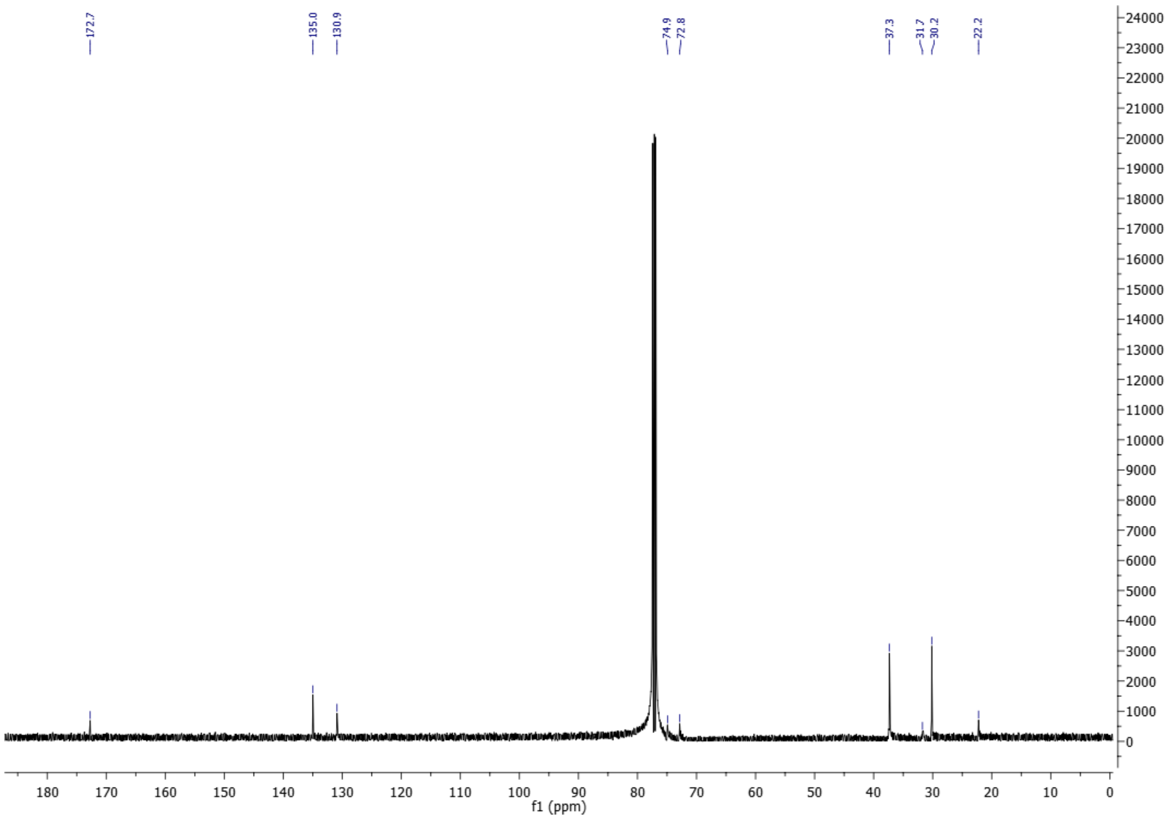

Figure S6. ^{13}C NMR spectrum of trucantenolide, **1** ($CDCl_3$, 100 MHz).

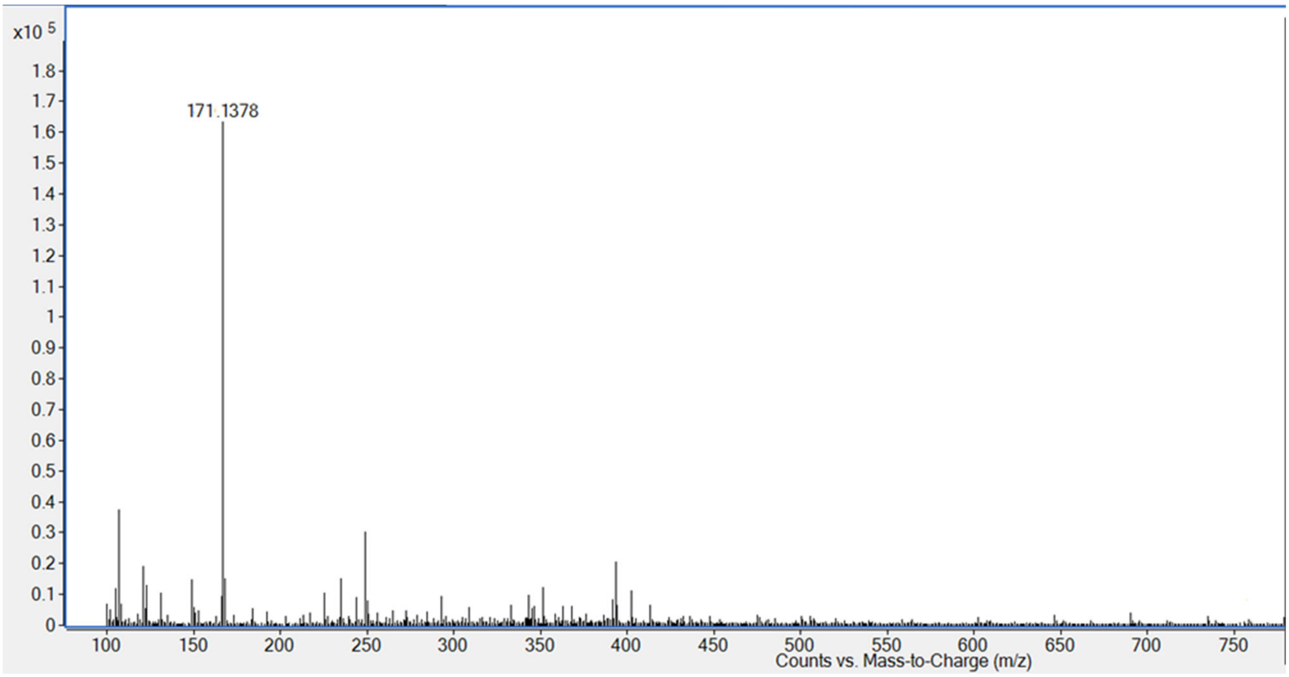

Figure S7. HRESI MS spectrum of of trucantenolide, **1** recorded in positive modality.

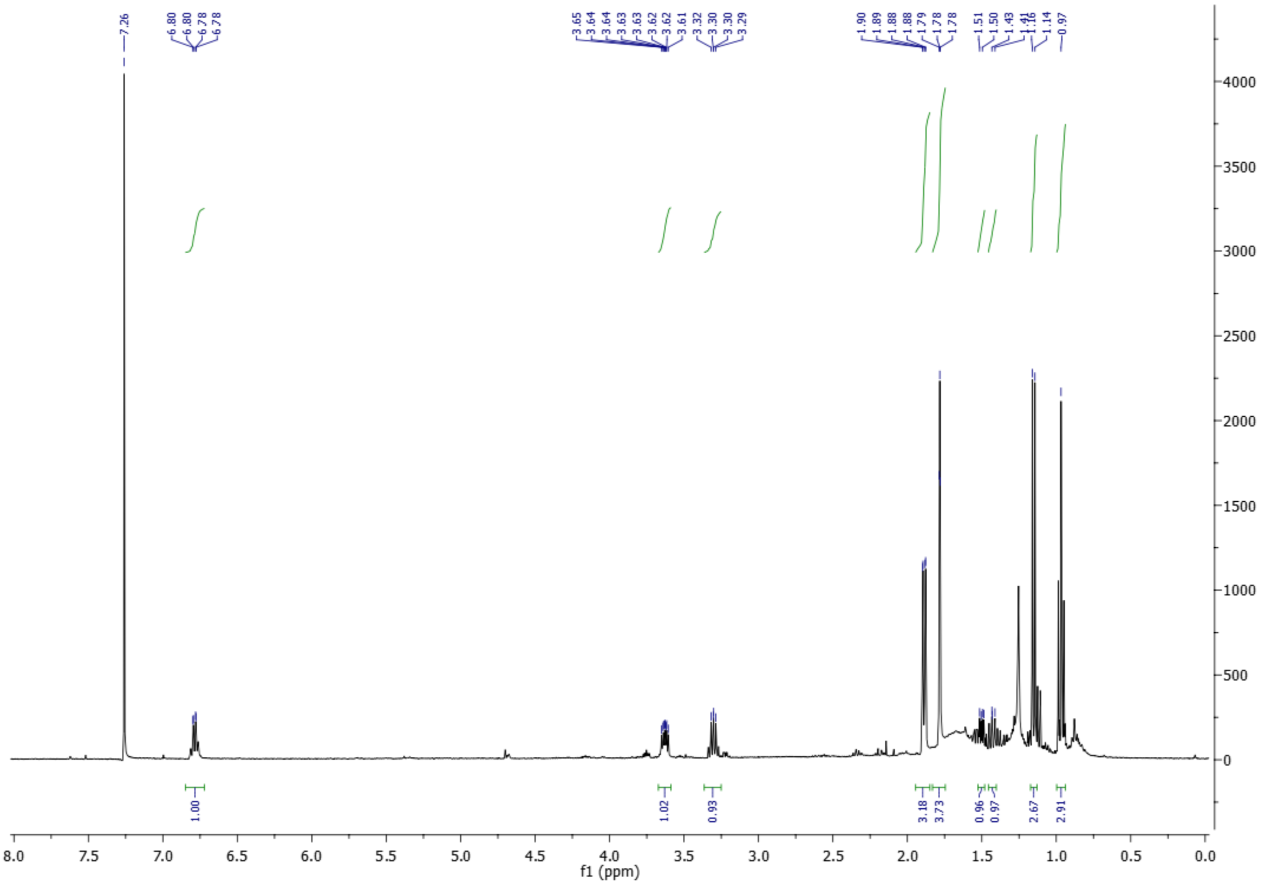

Figure S8. ^{1}H NMR spectrum of truncatenone, **2** ($CDCl_3$, 400 MHz).

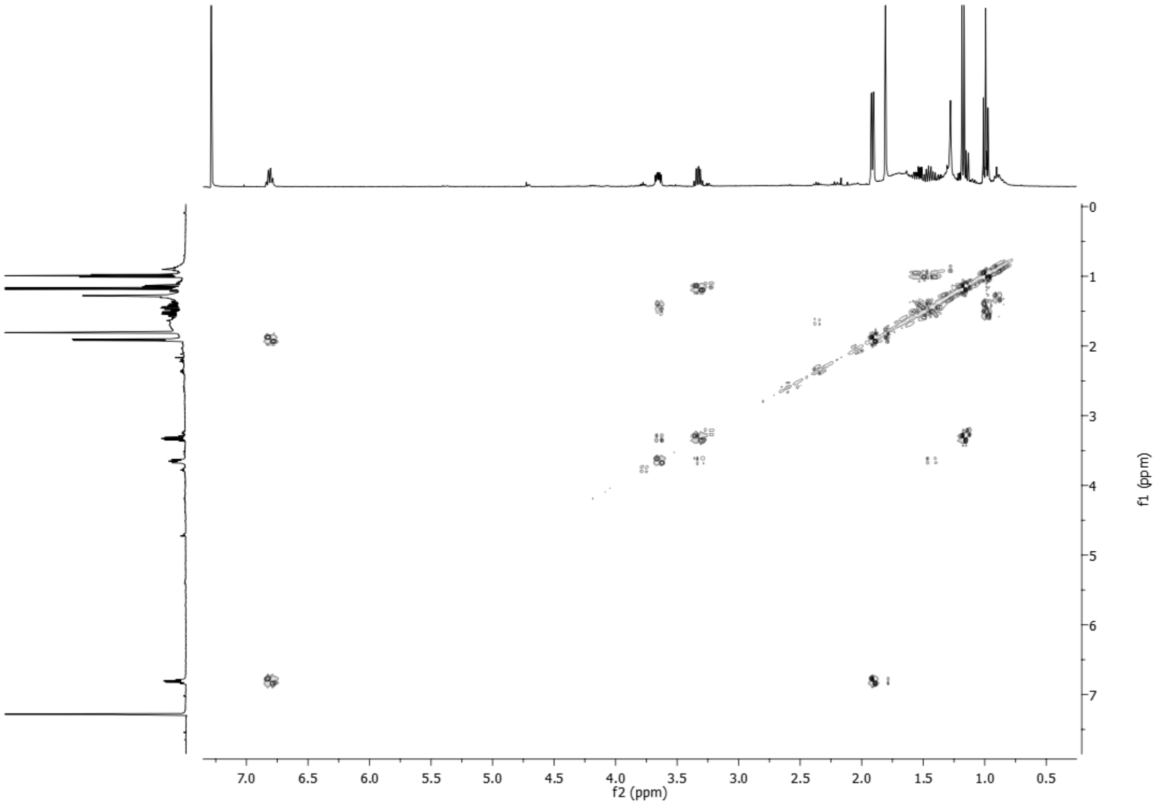

Figure S9. COSY spectrum of truncatenone, **2** ($CDCl_3$, 400 MHz).

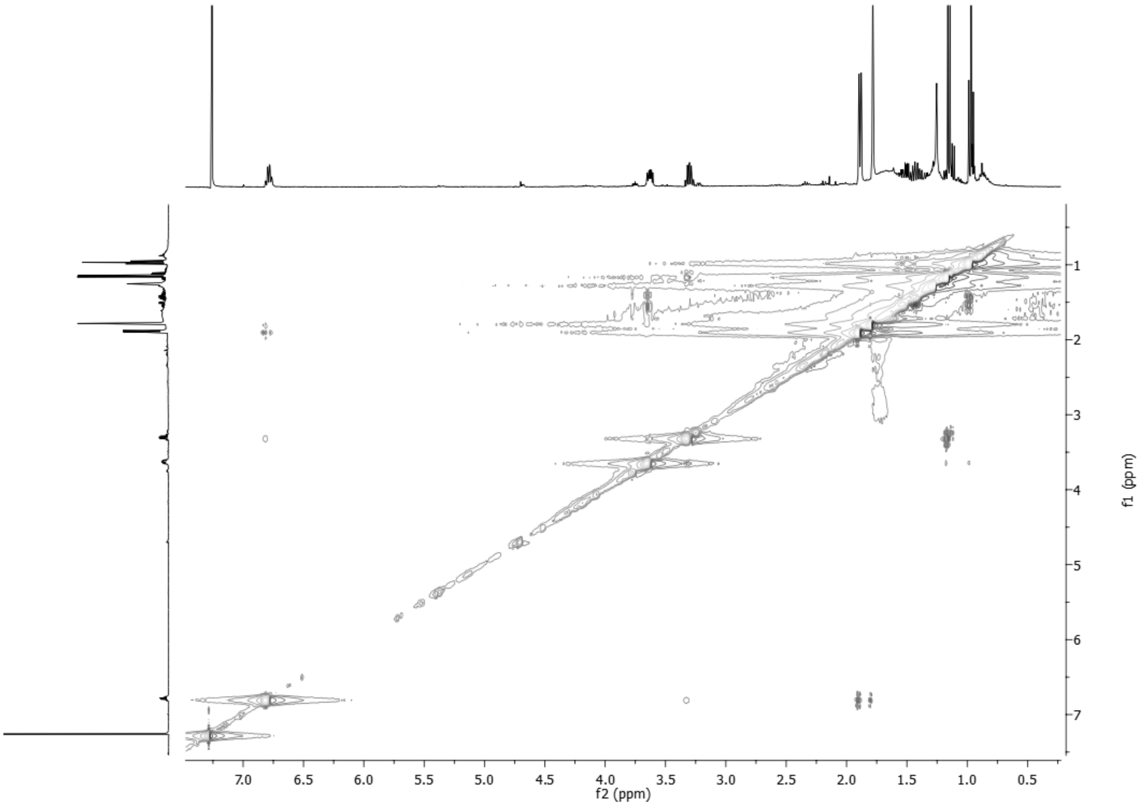

Figure S10. NOESY spectrum of truncatenone, **2** ($CDCl_3$, 400 MHz).

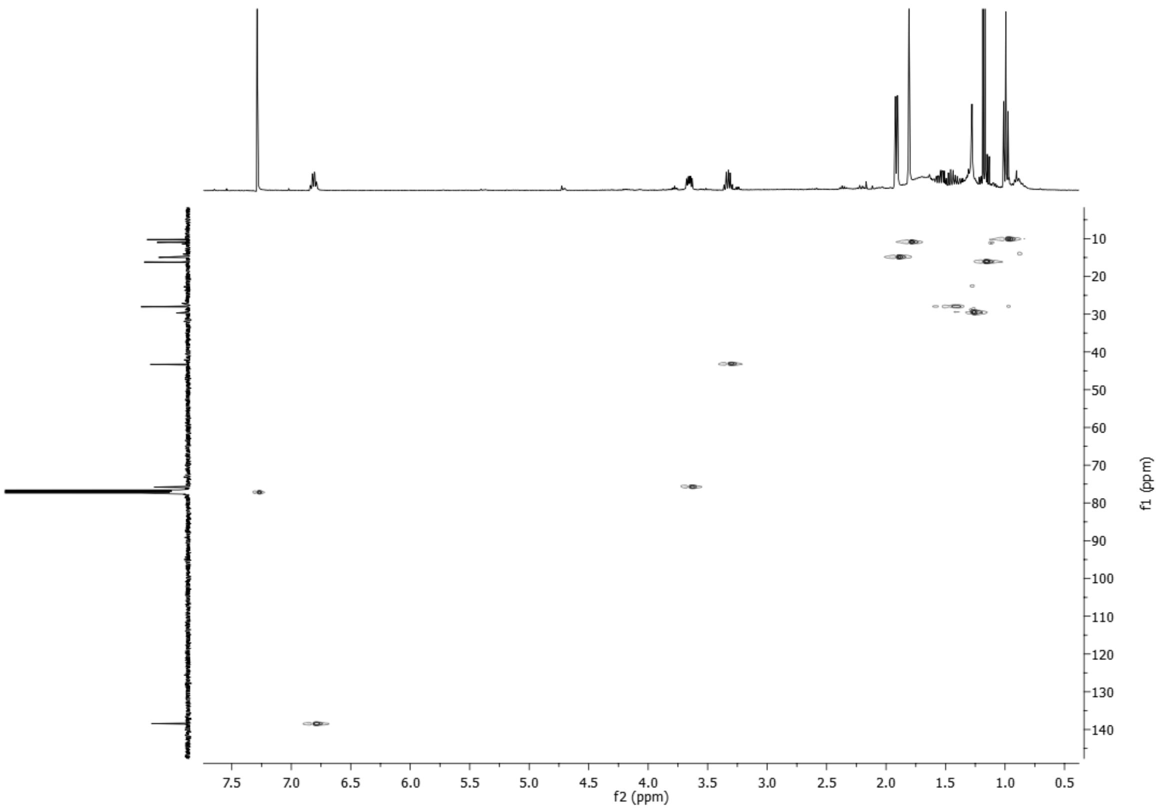

Figure S11. HSQC spectrum of truncatenone, **2** ($CDCl_3$, 400/100 MHz).

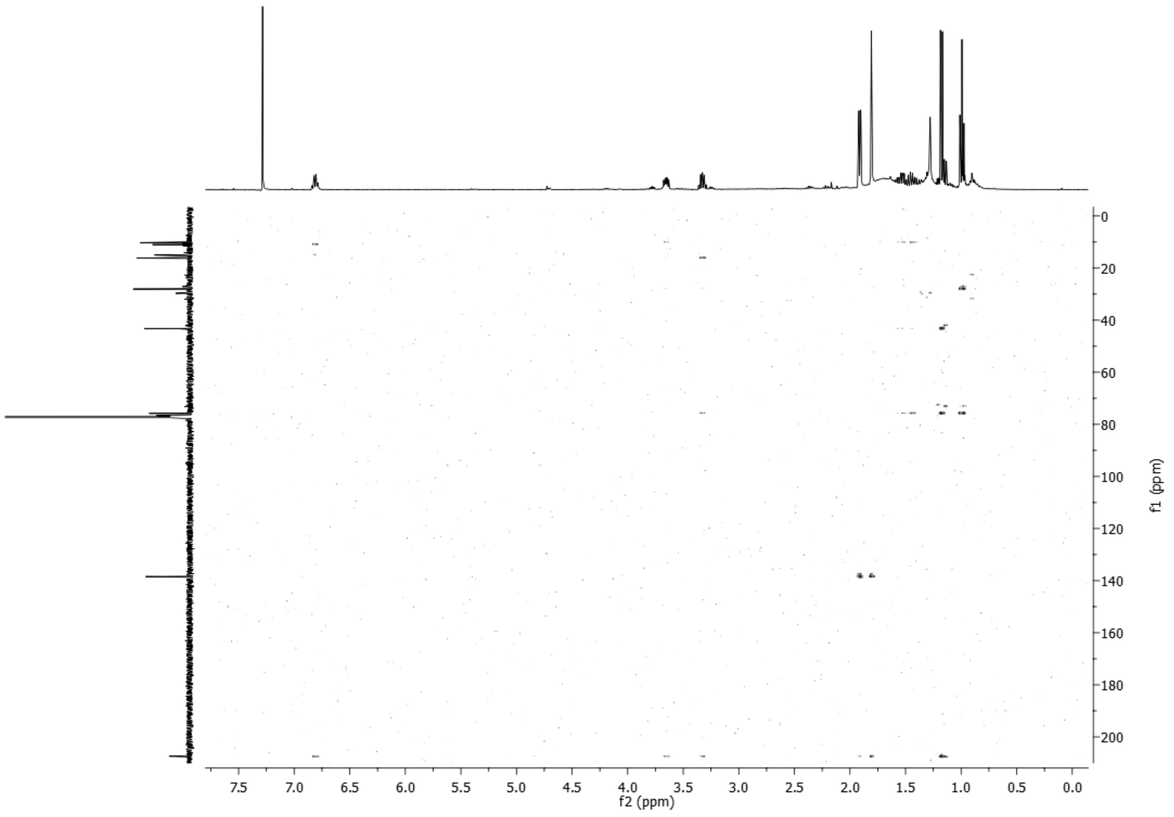

Figure S12. HMBC spectrum of truncatenone, **2** ($CDCl_3$, 400/100 MHz).

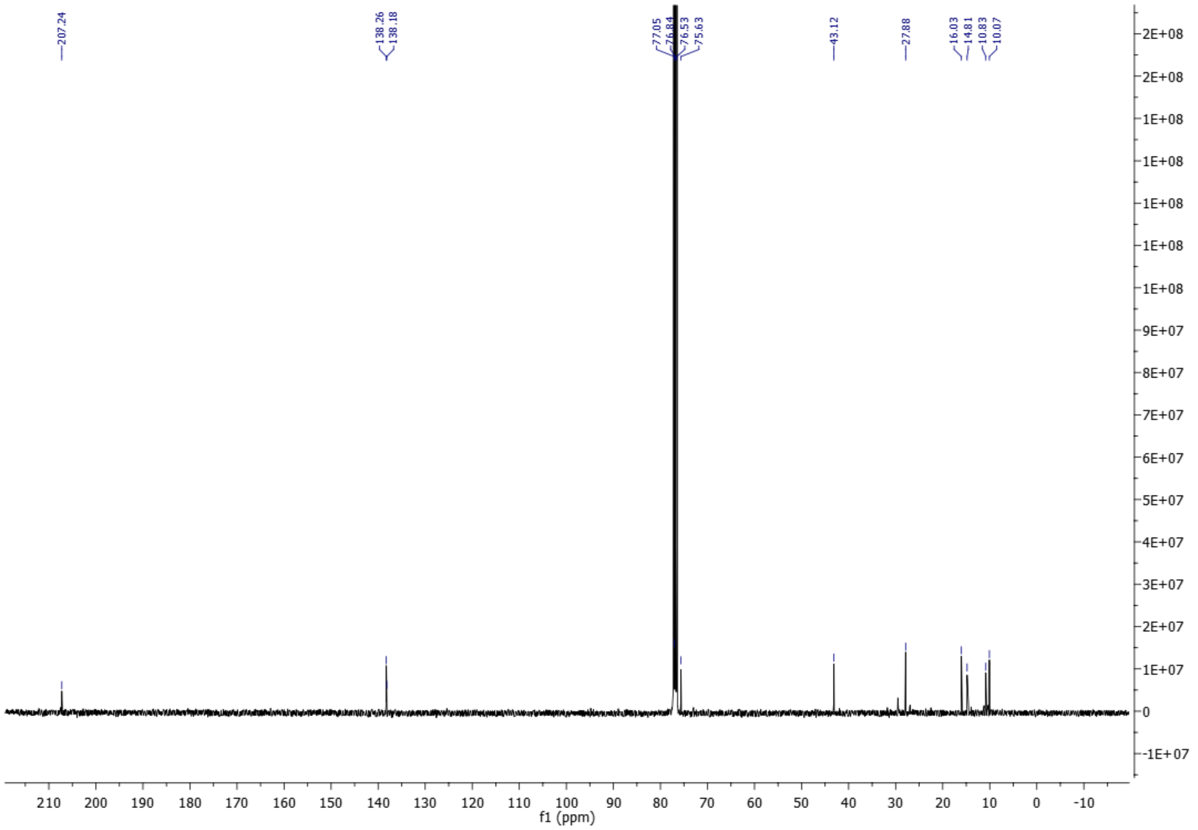

Figure S13. ^{13}C NMR spectrum of truncatenone, **2** ($CDCl_3$, 100 MHz).

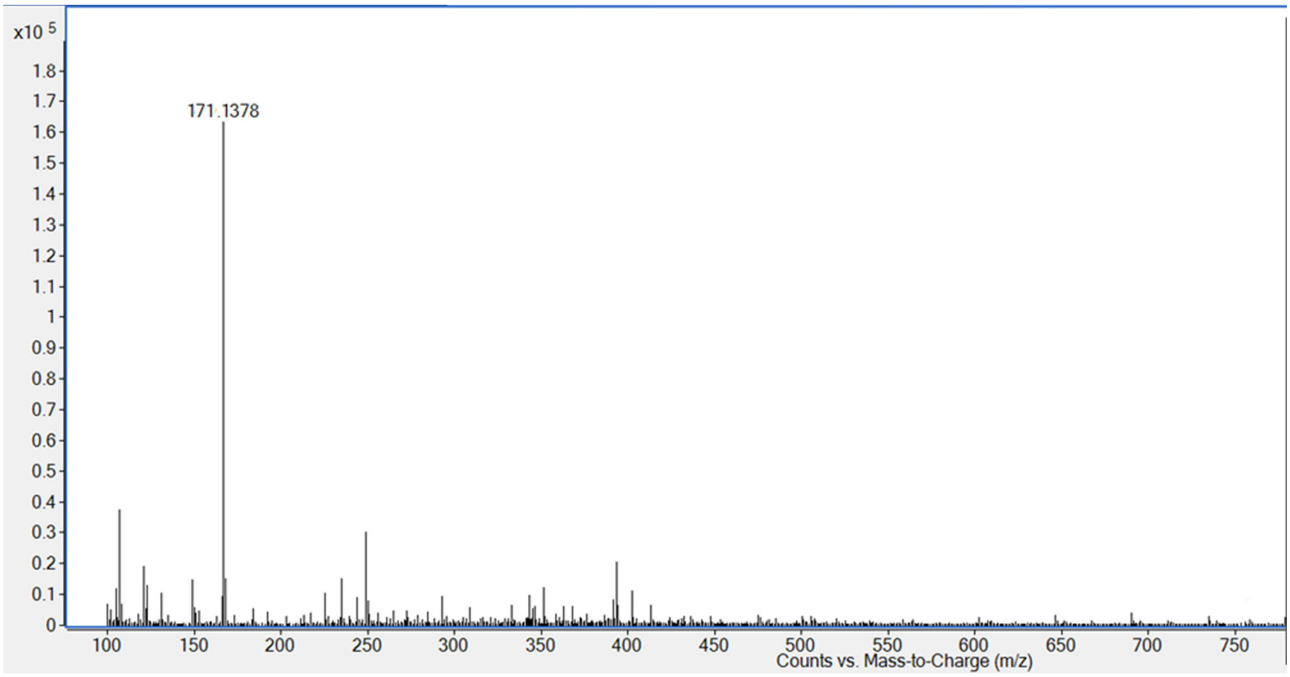

Figure S14. HRESI MS spectrum truncatenone, **2** recorded in positive modality.

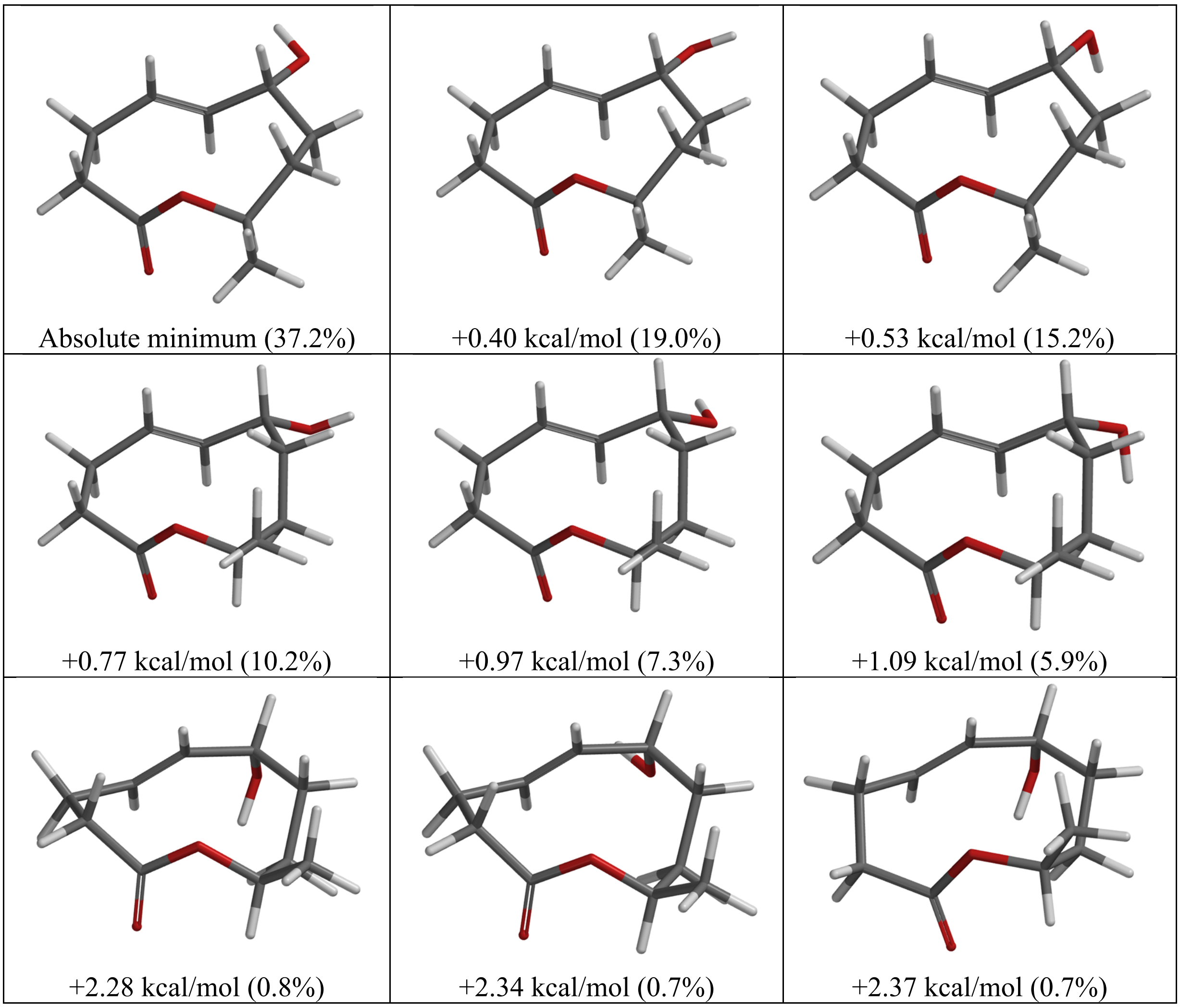

Figure S15. Low-energy conformers, relative internal energy and Boltzmann population at 300K calculated for compound (7*R*,10*R*)-**1** at ωB97X-D/6-311+G(d,p) level, including the Solvation Model based on Density (SMD) for acetonitrile.

Table S1. Summary of DP4+ test on (7*R*,10*S*)-**1** (isomer 1) and (7*R*,10*R*)-**1** (isomer 2). See Computational Section (main text) for details.

Functional		Solvent?		Basis Set		Type of Data	
B3LYP		Gas Phase		6-31G(d)		Scaled Shifts	

		DP4+	-	-	-	-	-
Nuclei	sp2?	Experimental	Isomer 1	Isomer 2	Isomer 3	Isomer 4	Isomer 5
C	x	172.7	173.6	173.2			
C		37.3	36.4	36.4			
C		30.2	31.4	31.6			
C	x	130.9	127.6	131.6			
C	x	135	137.6	137.1			
C		74.9	69.6	75.6			
C		37.3	34.9	36.4			
C		31.7	27.7	31.4			
C		72.8	71.6	72.6			
C		22.2	21.2	21.6			

	Isomer 1	Isomer 2
sDP4+ (H data)	-	-
sDP4+ (C data)	0.00%	100.00%
sDP4+ (all data)	0.00%	100.00%

Table S2. Summary of DP4+ test on (5*R*,6*S*)-**2** (isomer 1) and (5*S*,6*S*)-**2** (isomer 2). See Computational Section (main text) for details.

Functional		Solvent?		Basis Set		Type of Data	
B3LYP		Gas Phase		6-31G(d)		Scaled Shifts	

		DP4+	-	-	-	-	-
Nuclei	sp2?	Experimental	Isomer 1	Isomer 2	Isomer 3	Isomer 4	Isomer 5
C		14.8	17.3	16.8			
C	x	138.3	145.0	143.6			
C	x	138.2	134.6	135.3			
C		10.8	22.4	22.3			
C	x	207.2	212.4	212.0			
C		43.1	45.7	47.4			
C		16	11.1	16.1			
C		75.6	72.1	74.4			
C		27.9	27.7	28.3			
C		10.1	12.3	11.7			

	Isomer 1	Isomer 2
sDP4+ (H data)	-	-
sDP4+ (C data)	0.13%	99.87%
sDP4+ (all data)	0.13%	99.87%
